# Ultra-performance liquid chromatography-quadrupole time-of-flight mass spectrometry metabolomic profiling reveals harvest age dependent changes in the roots of *Pelargonium sidoides* DC

**DOI:** 10.1007/s11306-026-02505-6

**Published:** 2026-07-08

**Authors:** Kundani Khameli, Muinat Nike Lewu, Takalani Mulaudzi, Oluwafemi Ayodeji Adebo, Oluwatobi Samuel Oluwafemi

**Affiliations:** 1 Centre for Innovative Food Research (CIFR), Department of Biotechnology and Food Technology, Faculty of Science, University of Johannesburg, P.O. Box 17011, Doornfontein Campus, Johannesburg, 2028 South Africa; 2 Centre for Nanomaterials Science Research, Department of Chemical Sciences, Faculty of Science, University of Johannesburg, Doornfontein Campus, P.O. Box 17011, Johannesburg, 2028 South Africa; 3 Soil and Water Science Programme, Agricultural Research Council Infruitec-Nietvoorbij, Private Bag X5026, Stellenbosch, 7599 South Africa; 4 Department of Biotechnology, University of the Western Cape, Life Sciences Building, Private Bag X17, Bellville, 7535 South Africa

**Keywords:** *Pelargonium sidoides*, Metabolites, Cultivation, Harvest age, Coumarins

## Abstract

**Introduction:**

Cultivation of medicinal plants provides an opportunity for economic gain and health care accessibility. Ensuring consistent quality plant material is important for plants such as *Pelargonium sidoides* DC used for production of phytomedicines that are available in local and international markets. There is limited research on how agronomic factors affects phytochemicals in *P. sidoides* roots.

**Objective:**

To evaluate the effect of different irrigation regimes and harvesting age on metabolite accumulation in dried roots of *P. sidoides*.

**Methodology:**

Irrigation was applied at 75%, 50% and 25% plant available water (PAW) corresponding to well- watered, moderate water deficit and severe water deficit respectively. *P. sidoides*, which were harvested at 6, 12 and 18 months after imposing the different water deficit treatments, roots dried and subjected to ultra-performance liquid chromatography -quadrupole time-of-flight mass spectrometry (UPLC-QTOF-MS) coupled with multivariate statistical analysis.

**Results:**

Unsupervised multivariate analysis showed that irrigation did not affect the obtained metabolic features. Orthogonal partial least squares discriminant analysis (OPLS-DA) showed statistically significance difference between 6 versus 12- and 18- months harvest ages. Compounds such as umckalin, epigallocatechin dimer, and gallic acid were increased in 12 and 18 months compared to the 6 months harvest. Sucrose/trehalose was increased by 0.2431- and 0.560- fold in 6 months compared to 12- and 18-months harvest ages respectively. On quantification, umckalin increased from 260.40 mg/kg DW at 6 months to 431.61 mg/kg DW at 12 months, while epigallocatechin rose from 342.98 mg/kg DW to 505.58 mg/kg DW over the same period. Umckalin sulphate was highest at 6 months under 25% PAW (7110.32 mg/kg DW) and decreased at 12 months harvest under the same irrigation level (4522.05 mg/kg DW).

**Conclusion:**

The results indicated that younger plants accumulated more primary metabolites and modified secondary metabolites, whereas older plants accumulate more secondary metabolites. Moreover, cultivation of *P. sidoides* in limited water is plausible.

**Supplementary Information:**

The online version contains supplementary material available at https://doi.org/10.1007/s11306-026-02505-6.

## Introduction

Herbal traditional medicine is widely used globally, with approximately 80% of populations in developing countries depending on it for their primary health care (Hlatshwayo et al., 2025). The use and commercialisation of these medicinal plants is often guided by factors such as limited access to health care, high cost of western medicine, and education. While commercialisation provides economic gain to rural communities it poses a threat to the biodiversity and sustainability of these plants through overharvesting (Ndhlovu et al., 2025). Cultivating medicinal plants therefore serve as a vital solution to conserve the natural medicinal plant populations and meet the global market demand (Zhong et al., 2025). Harvesting medicinal plant at their optimal conditions is important to maintain high quality and quantity of bioactive compounds. Plant age, although this varies with plant species is one of the factors to consider when optimizing medicinal plants (Hazrati et al., 2024).

Plants water requirement also differs among plant species. With the agriculture sector being the largest consumer of water worldwide, it is prominent to investigate the water use of plants to improve water use efficiency, growth and sustainable agricultural practices (Ingrao et al., 2023). The need to study the water use of plants has become more prominent especially in South Africa where semi-arid and dry regions have already started to face water shortages caused by climate change (Olatoye & Fru, 2025). Additionally, drought and water scarcity are estimated to intensify by the end of century (Mofokeng et al., 2020; Msweli et al., 2025). Applying different irrigation ranging from well-watered to severe water deficit conditions provides an effective way to evaluate the plants responses to limited water availability, exploring water deficit as a potential strategy for sustainable water use and conservation of the species (Mofokeng et al., 2015; Xing & Wang, 2024). Integrating plant age and water requirement is therefore essential for not only optimising phytochemical production, but also increasing water use efficiency, and promoting conservation of plants.

Plant metabolomics is an emerging valuable tool in medicinal plants, offering comprehensive analysis of their phytochemical constituents. It uncovers the plants primary and secondary metabolite composition, its pharmacological potential and further provides qualitative and quantitative plants response to environmental and developmental factors (Alum et al., 2025; Hao et al., 2025). Analytical techniques used in plant metabolomics includes, Nuclear magnetic resonance (NMR), gas chromatography–mass spectrometry (GC–MS), and liquid chromatography–mass spectrometry (LC–MS) (Mutha et al., 2025). The use of these analytical techniques together with multivariate statistical analysis aids in identifying metabolic markers, distinguishing chemotypes, and quantifying changes in metabolites composition in response to external factors. This approach offers a better understanding into the plant’s biosynthetic pathways and further facilitate the optimisation of environmental and agronomic conditions for the cultivation of plants (Alum et al., 2025; Głuchowska et al., 2025).

*Pelargonium sidoides* DC is a medicinally important plant, native to the southern Africa. It has been used for centuries in traditional medicine to treat ailments such as respiratory infections, diarrhoea, and colic (Brenes et al., 2024; Khameli et al., 2026; Moyo & Van Staden, 2014). Pharmacological studies through in vivo and in vitro studies have reported the plants antiviral, antibacterial, anti-inflammatory, immunomodulatory and most recently protective effects (Abu-Zahra et al., 2025; Reina et al., 2024). Additionally, clinical trial effectiveness and safety have been reported for use in common cold, acute bronchitis and tonsillopharyngitis in both children and adults (Careddu & Pettenazzo, 2018). The plants extract has been developed into several phytomedicines available commercially, hence its economic significance is undeniable as its products are part of local and international market (Moyo & Van Staden, 2014; Mtimkulu et al., 2024).

There have been reports on localised declining wild populations of *P. sidoides* of which are associated with overharvesting, which is further attributed to the high demand of the plant for local and international use (Mofokeng et al., 2020; Moyo & Van Staden, 2014). With the plant’s slow regeneration rate, increasing phytomedicines developed from the plant, its availability is at risk (Motjotji, 2011; Mtimkulu et al., 2024). Additionally, although according to our knowledge no specific reports have been done on the direct influence of climate change to *P. sidoides*, climate changes such as drought, higher temperatures, and carbon dioxide affect the growth, development and production of secondary metabolites in plants (Jangpangi et al., 2025). Despite its therapeutic significance, economic viability and environmental changes there is limited research on how cultivation factors such as irrigation and harvest age influence its metabolite composition. Understanding these effects is essential for establishing sustainable cultivation practices that optimise the production of bioactive compounds. Thus, the aim of this study is to use ultra performance quadrupole time of flight mass spectrometry (UPLC-QTOF-MS) based metabolomic profiling to investigate the effects of different irrigation regimes and harvest ages on the metabolic composition of *P. sidoides*.

## Materials and methods

### Cultivation and sampling

*Pelargonium sidoides* plant materials (sprouted root tubers) were obtained from the Agricultural Research Council – Vegetable, Industrial and Medicinal Plants (ARC-VIMP) gene bank, under accession number M2009/38. The trial was carried out in a tunnel at the Agricultural Research Council Infruitec-Nietvoorbij research facility in Stellenbosch, South Africa (33°54′52.38″S, 18°51′40.27″E). Plants were established in pots and maintained under uniform conditions for seven (7) months prior to treatment application. A randomised block design with four replications was employed, and irrigation was administered using a drip system. Three distinct irrigation regimes were implemented 25%, 50%, and 75% of plant-available water (PAW), representing severe water deficit, moderate water deficit, and well-watered treatment, respectively. Plants were harvested after 6, 12, and 18 months of treatment. The harvested plant root materials were oven dried, powdered and stored.

### Chemicals and reagents

LC-MS grade methanol, acetonitrile, analytical-grade formic acid (≥ 98%) and reference standards (umckalin and epigallocatechin) were purchased from Sigma-Aldrich (St. Louis, MO, USA). Ultrapure water was produced using a Milli-Q water purification system (Millipore, Billerica, MA, USA). Leucine enkephalin and sodium formate were procured from Waters Corporation (Milford, MA, USA).

### Sample preparation

About 0.25 g of dried, ground plant material was weighed into a 50 mL Falcon centrifuge tube and extracted in 25 mL of 100% methanol. The samples were vortexed briefly and then sonicated in an ultrasonic bath (0.5 Hz, Integral Systems, RSA) for 60 min at room temperature (25 °C ± 1). Following extraction, the samples were centrifuged (Hermle Z160M, Hermle Labortechnik GmbH, Wehingen, Germany) at 3000×*g* for 5 min, the supernatants collected, and aliquots were transferred into glass vials for UPLC-QTOF-MS analysis.

### Instrumental analysis

A Waters Cyclic Quadrupole Time-of-Flight (qTOF) mass spectrometer (Waters, Milford, MA, USA), combined with Waters Acquity Ultra-Performance Liquid Chromatograph (UPLC), was used for high-resolution UPLC-MS analysis. Before entering the mass spectrometer, the effluent from the column passed through a Photodiode Array (PDA) detector, for simultaneous acquisition of UV and MS spectra. Electrospray ionization (ESI) was conducted in negative ion mode under the following conditions: capillary voltage of 2.5 kV, cone voltage of 15 V, desolvation (temperature of 275 °C, gas flow at 650 L/h), and source temperature of 120 °C. MS data were acquired in resolution mode over an *m/z* range of 100 to 1500. Data acquisition was conducted in MSE mode, where two channels of MS data were simultaneously recorded, one at low collision energy (4 V) and the other using a collision energy ramp from 40 to 100 V for generation of fragmentation spectra. Leucine enkephalin was used as the lock mass for real-time correction, while sodium formate was employed for instrument calibration.

Chromatographic separation was done using a Waters HSS T3 column (2.1 × 150 mm, 1.7 μm). The mobile phase was water with 0.1% formic acid (solvent A) and acetonitrile with 0.1% formic acid (solvent B). The elution gradient started with a 1-minute at 100% A, followed by a linear increase to 28% B over a duration of 11 min, then increased to 40% B over 50 s. This was followed by a 1.5-minute wash step at 100% B and re-equilibration to initial conditions for 2 min. The flow rate was 0.3 mL/min, with an injection volume of 0.5 µL, and a column temperature at 60 °C.

### Data processing, compound identification and multivariate analysis

MSE data were processed using MS-DIAL, where Function 1 (low energy) and Function 2 (high energy) were used to generate MS¹ and MS² spectra, as well as extracted ion chromatograms with associated peak height intensities. Each aligned feature was exported to MS-FINDER for identification of the compounds. Based on accurate mass and elemental composition, candidate structures were retrieved from curated databases and subjected to in-silico fragmentation. The resulting theoretical spectra were matched against experimental MS² data, and a confidence score (0–10) was assigned; only candidates with scores of ≥ 6 that were either previously reported in *P. sidoides* or biochemically plausible for the species were retained as tentative identifications. Compound identifications were then assigned metabolomics standards initiative (MSI) classification confidence levels adapted from Fiehn et al. (2007) and Schymanski et al. (2014), ranging between level 1 (most confident) and level 4 (least confident). Level 1 was used for compounds verified using authentic reference standards. Level 2a was provided to those corresponding to matches against spectral database entries having high similarity, while level 2b was used to describe identification supported by literature reports of *P. sidoides* analysed by LC-MS. Level 3 was provided for tentative identification supported by MS/MS fragmentation data where structure information was available but positional isomers could not be ruled out. Level 4 was assigned when only the molecular formula could be determined with confidence. Biological replicates analysed were subjected to multivariate statistics, principal component analysis (PCA) and OPLS-DA were performed using SIMCA software Version 18 (Sartorius, Umeå, Sweden). OPLS-DA–derived raw p-values were subjected to false discovery rate (FDR) correction using the Benjamini–Hochberg (BH) procedure in RStudio (version 2026.01.1+403).

### Semi-quantification and statistical analysis

Umckalin standard curve (Fig. S1a) with R^2^ = 0.9912, with limit of detection (LOD) of 1.2533 ppm and limit of quantification (LOQ) of 3.7979 ppm was used as a reference compound. Due to absence of other compounds, this was used to provide a semi-quantitative estimation of structurally related coumarin derivatives, including umckalin sulphate, isofraxidin, and dihydroxy coumarin sulphate, with concentrations expressed as umckalin equivalents. Similarly, epigallocatechin was employed as a reference standard (Fig. S1b), having an R^2^ = 0.9976, LOD = 10.1234 ppm, and LOQ = 30.6770 ppm to quantitatively estimate concentrations of related flavan-3-ols, including gallocatechin, epigallocatechin dimer, and gallocatechin dimer, which were reported as epigallocatechin equivalents. For each treatment group, four biological replicates were analysed, and the average of these replicates was used to represent the treatment value. Statistical analyses were performed using Analysis of Variance (ANOVA) with the General linear model (GLM) procedure in SAS version 9.4 (SAS Institute Inc., Cary, NC, USA). Treatment means were compared using the least significant difference (LSD) test at a significance level of 5% (*p* = 0.05), and differences with p-values less than or equal to 0.05 were considered statistically significant.

## Results and discussions

### Metabolites identification

The untargeted UPLC-QTOF-MS screening resulted in 173 features (Table S1), due to lack of authentic standards only 26 were identified as distinct metabolites. Eleven of identified compounds were coumarins and their derivatives. Among these, Umckalin (*m/z* 221.04597, 6.847 min), a recognised marker of *P. sidoides* was identified. This finding aligns with earlier studies that have identified and reported umckalin as a marker compound in *P. sidoides* roots (Brendler et al., 2024; Hauer et al., 2010; Viljoen et al., 2015). Its sulphated form, umckalin sulphate (*m/z* 301.00244, 6.001 min), was also identified. Additional coumarins, including scopoletin (*m/z* 191.03526, 5.007 min), fraxin (*m/z* 369.0834, 4.547 min), and fraxidin (*m/z* 221.04573, 6.053 min) were tentatively identified, however the possibility of it being fraxinol cannot be ruled out as they are isomers (Brendler et al., 2024). Sulphated coumarins, including a dihydroxy coumarin sulphate, scopoletin sulphate, and isofraxidin sulphate, were also identified. The presence of these coumarins and their sulphates have been previously described in *P. sidoides* roots (Hauer et al., 2010; Kolodziej, 2007).

Flavonoids constituted another major group identified in the roots of *P. sidoides*, this group included epigallocatechin, epicatechin, and their oligomers (dimers and trimers), consistent with earlier reports of flavonoids in *P. sidoides* roots (Jekabsone et al., 2019; Panara et al., 2022). While epigallocatechin and gallocatechin are structural isomers their successful tentative identification was aided by a standard compound of epigallocatechin. As in this study, eriodictyol has been previously identified in *P. sidoides*, this study also confirmed the presence of its sulphated form, eriodictyol sulphate, adding to the sulphated metabolites in *P. sidoides*. In addition, two turgorins (leaf movement factor 3 and leaf movement factor 2) were annotated, which had been previously reported in *P. sidoides* by (Brendler et al., 2024). A disaccharide was annotated as sucrose/trehalose (*m/z* 341.10965, 1.226 min), representing a common primary metabolite in the roots of *P. sidoides*. The presence of trehalose has been previously reported by Panara et al. (2022) (Table 1).

**Table 1:** Compounds tentatively identified in *P. sidoides* root samples

RT	*m/z*	MS/MS fragment ions	Chemical formula	Metabolite annotation	MSI classification level
*Coumarins and derivatives*					
3.963	272.97141	193.01424, 272.97156	C_9_H_6_O_8_S	Dihydroxy coumarin sulphate	Level 3
4.547	369.08340	369.08304	C_16_H_18_O_10_	Fraxin	Level 2a
4.910	270.99237	145.93121, 174.95584, 191.03503, 206.97221, 235.92664, 270.99231	C_10_H_8_O_7_S	(6-methoxy-2-oxo-chromen-7-yl) hydrogen sulphate (Scopoletin sulphate)	Level 2a
5.204	301.00241	301.00070	C_11_H_10_O_8_S	Isofraxidin sulphate	Level 3
5.813	316.99716	206.99339, 316.99704	C_11_H_10_O_9_S	8 hydroxy 5.7 dimethoxycoumarin-6-sulphate	Level 2b
5.007	191.0353	191.03526	C_10_H_8_O_4_	Scopoletin	Level 2a
6.053	221.04573	145.93227, 174.95686, 190.99886, 206.02228, 221.04585	C_11_H_10_O_5_	Isofraxidin/fraxinol	Level 2b
6.001	301.00244	163.00377, 164.00728, 190.99869, 192.00214, 193.00455, 206.02243, 221.04587, 222.04961, 301.00238	C_11_H_10_O_8_S	Umckalin sulphate	Level 2b
4.930	207.03040	192.00648, 207.02992	C10H8O5	Fraxetin	Level 2a
6.162	237.04060	179.00008, 206.99387, 207.9977, 222.01741, 223.02235, 237.04065,	C_11_H_10_O_6_	6.8-dihydroxy-5.7-dimethoxy coumarin	Level 2b
6.847	221.04597	163.00395, 190.99876, 192.0024, 206.02199, 221.04565	C_11_H_10_O_5_	Umckalin	Level 1
*Flavonoids*					
3.189	913.18549	305.06567, 423.073, 541.07617, 913.18152	C_45_H_38_O_21_	Epigallocatechin trimer	Level 3
3.296	609.12700	177.01987,241.00307, 243.03128, 255.02971, 273.04282, 283.02667, 303.05191, 305.06671	C_30_H_26_O_14_	Epigallocatechin dimer	Level 3
3.383	913.18378	305.06631, 423.07275, 727.12994, 913.1825	C_45_H_38_O_21_	Gallocatechin trimer	Level 3
3.476	305.06690	261.04227, 303.05075, 305.06717	C_15_H_14_O_7_	Gallocatechin	Level 3
3.567	609.12530	177.01944, 243.03038, 255.03018, 256.03632, 261.04062, 273.04031, 283.02454,	C_30_H_26_O_14_	Gallocatechin dimer	Level 3
3.947	305.06738	193.01353, 272.97165, 305.06644	C_15_H_14_O_7_	Epigallocatechin	Level 1
4.211	289.07210	272.97147, 289.07147	C_15_H_14_O_6_	Epicatechin	Level 2a
5.563	367.01312	367.01099	C_15_H_12_O_9_S	Eriodictoyl sulphate	Level 4
7.166	585.23438	328.13077, 329.10504, 341.10486, 344.12625, 359.14984, 371.15005,415.18027, 507.2027	C₂₈H₂₆O₁₆	Galloyl-apigenin-C-hexoside	Level 3
5.490	287.05640	259.0614,269.04529, 287.05661	C_15_H_12_O_6_	Eriodictyol	Level 2a
*Phenolic acid*					
3.024	169.01420	169.01474	C_7_H_6_O_5_	Gallic acid	Level 2a
*Phenolic glycosides*					
3.094	331.10373	303.05005, 305.06738, 331.10123	C_14_H_20_O_9_	Koaburaside	Level 2b
*Turgorins*					
2.911	328.04596	328.04547	C_10_H_12_N_5_O_6_P	Leaf movement factor 3 (LMF3)	Level 2b
2.963	344.04030	305.01797, 306.02176, 326.01346, 331.06644, 344.04056	C_10_H_12_N_5_O_7_P	Leaf movement factor 2 (LMF2)	Level 2b
*Sugar*					
1.226	341.10965	341.10919	C_12_H_22_O_11_	Trehalose/sucrose	Level 2a

*RT* Retention time, *m/z* mass to charge ratio, *MSI* metabolomics standards initiative, levels (1 = authentic standards, 2a = spectral databases, 2b = literature reports, 3 = ms/ms fragmentation data, 4 = molecular formulas)

### Multivariate analysis

#### Principal component analysis of UPLC-QTOF-MS data

**Fig. 1:**
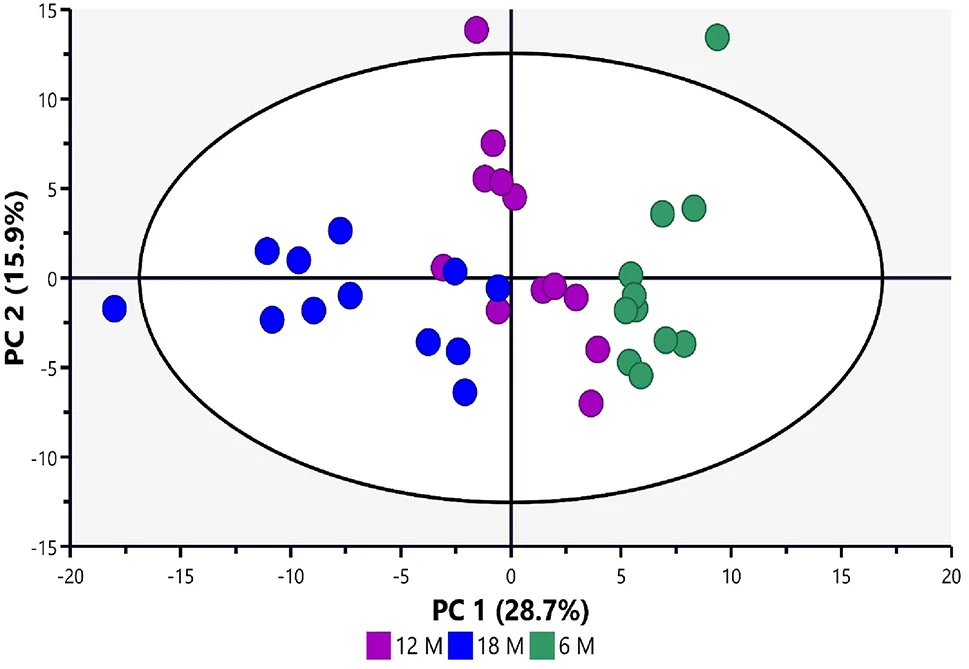
Principal component analysis scores plot showing the relationship between metabolite profiles of *P. sidoides* harvested at 6, 12, and 18 months, as obtained by UPLC-QTOF-MS

Unsupervised chemometrics using principal component analysis (PCA) was applied to UPLC-QTOF-MS data, with the first two principal components (PC1 and PC2) accounting for 28.7% and 15.9% of the total variance, respectively (Fig. 1). When the samples were visualised by irrigation treatment and the interaction of irrigation (Fig. S2a) and harvest age (Fig. S2b), no trend or separation was observed, suggesting that water availability within the tested range did not substantially affect the obtained root metabolites of *P. sidoides* roots. Consistent with these findings, Mofokeng et al. (2020) reported that unsupervised PCA of NMR metabolomics data assessing effect of irrigation and nitrogen supply on *P. sidoides* roots showed no clear separation of the samples. This robustness is plausible for a species of plants adapted for arid environments, for which inner processes of metabolism would be more stable to outside variations such as water availability (Bistgani et al., 2024; Lewu et al., 2007).

**Fig. 2:**
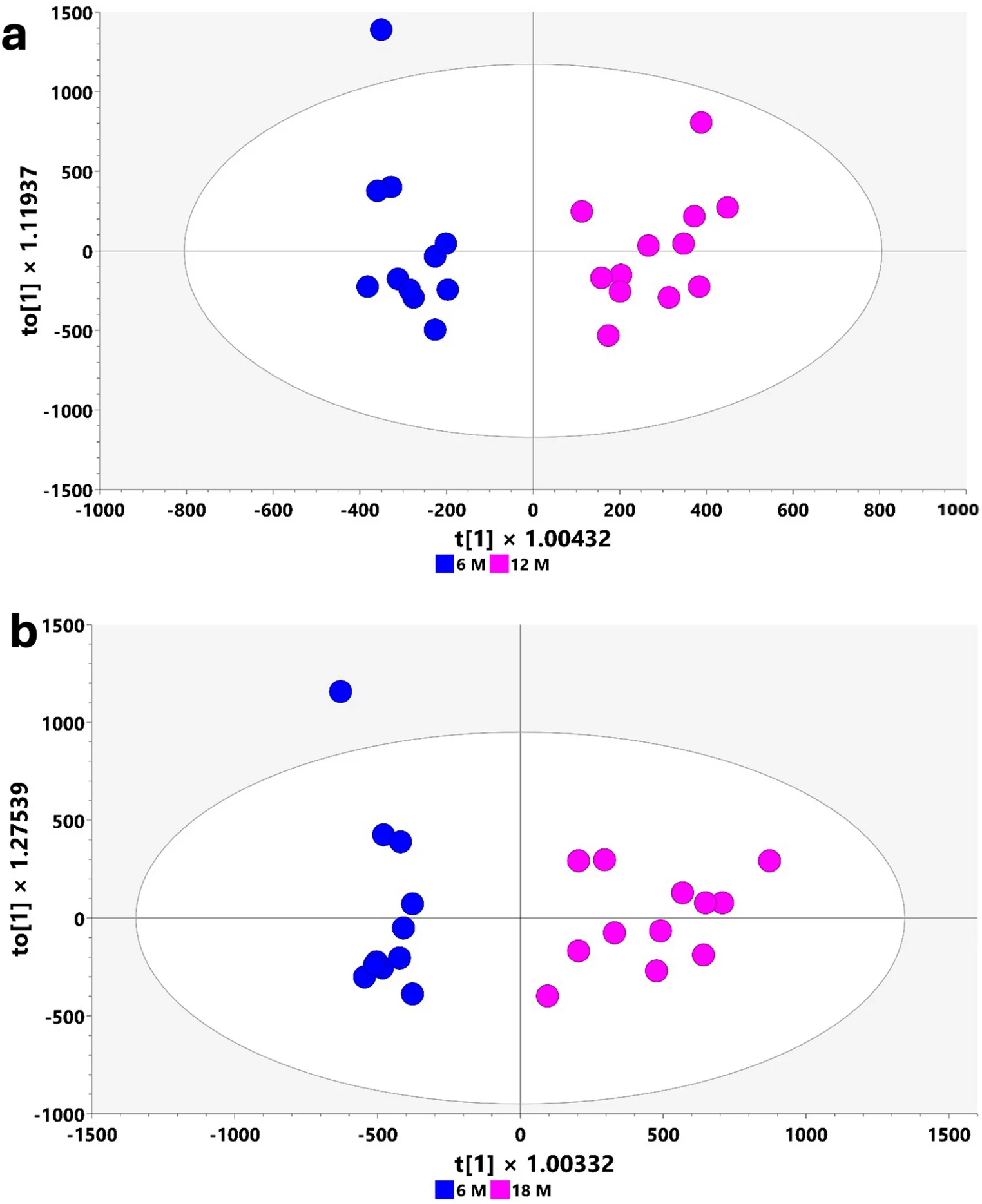
Supervised OPLS-DA score plots of *P. sidoides* metabolite profiles obtained by UHPLC-QTOF-MS: **a** comparison of 6-month (6 M) and 12-month (12 M) harvests with model performance R^2^Y(cum) = 0.908 and Q^2^(cum) = 0.849; **b** comparison of 6-month (6 M) and 18-month (18 M) harvests with R^2^Y(cum) = 0.881 and Q^2^(cum) = 0.814

However, the PCA analysis revealed a clear separation of samples based on the harvest ages (Fig. 2), particularly the 6-month samples clustered distinctly from the 12- and 18-month harvests. This clustering suggests that harvest age had a strong impact on the obtained metabolic profile of dried *P. sidoides* roots. This is most likely a consequence of ontogenetic variations in secondary metabolite accumulation, as observed in the OPLS-DA, where metabolites such as trehalose, sucrose, and galloyl-apigenin-C-hexoside were more abundant in younger roots (6 months harvest age), while isofraxidin/fraxinol, and an epigallocatechin dimer accumulated predominantly in older roots (12–18 months harvest age), which may be part of the plant’s developmental strategy. Similar age-related metabolites changes have been observed in other plants of medicinal importance, including ginseng berries (Park et al., 2019), *Andrographis paniculata* (Tajidin et al., 2019), and *Stellaria dichotoma L. var. lanceolata Bge*. (Li et al., 2023).

#### OPLS-DA of metabolite features across harvest ages

**Fig. 3:**
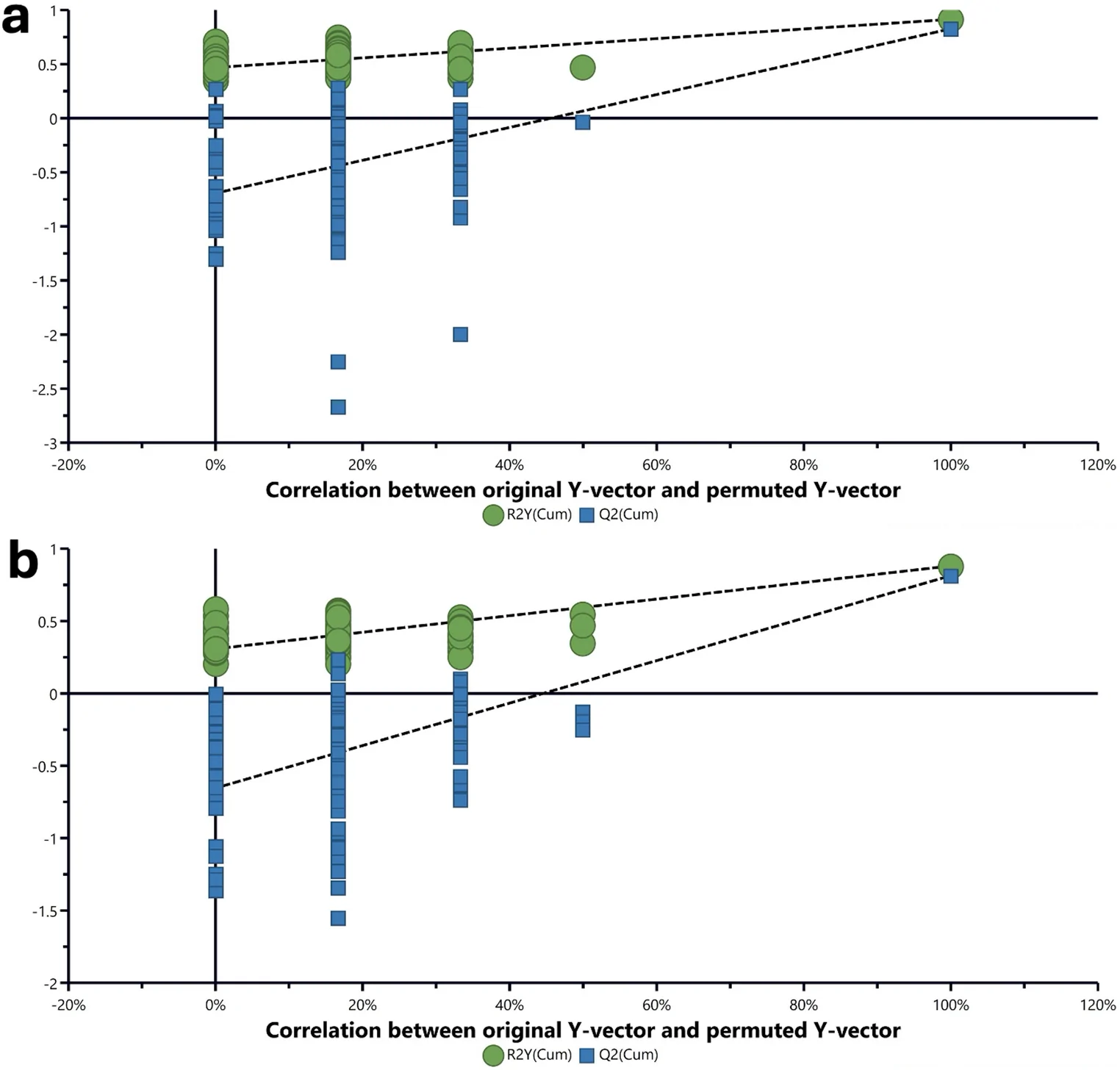
Permutation plots generated from the OPLS-DA model from UPLC-QTOF-MS data, with 100-permutation tests applied (**a** 6 versus 12 months; **b** 6 versus 18 months)

To further evaluate age-related metabolic differences observed in PCA, a supervised OPLS-DA models were applied. Clear discrimination was observed between 6-month and 12-month harvests (Fig. 2a), as well as between 6-month and 18-month harvests (Fig. 2b), indicating strong age-dependent metabolic variation in *P. sidoides* roots. The 6 vs. 12-month model showed strong explanatory and predictive performance (R^2^Y = 0.908; Q^2^ = 0.849), while the 6 vs. 18-month model also demonstrated robust model quality (R²Y = 0.881; Q^2^ = 0.814). Model validity was confirmed using permutation testing (*n* = 100), where the permuted models (Fig. 3) yielded substantially lower R²Y intercepts and negative Q² values (6 versus 12 months: R^2^Y = 0.55, Q^2^ = −0.434; 6 versus 18 months: R²Y = 0.334, Q^2^ = −0.366), indicating that the original models were not the result of random correlation.

Statistical significance was further supported by CV-ANOVA (6 versus 12 months: *p* = 1.225 × 10⁻⁵; 6 vs. 18 months: *p* = 1.015 × 10⁻⁶), confirming model robustness. The 6 vs. 12-month separation was modelled using one predictive and two orthogonal components (1 + 2+0), while the 6 vs. 18-month model utilised one predictive and one orthogonal component (1 + 1 + 0). While PCA demonstrated clear unsupervised clustering trends, the supervised OPLS-DA models allowed for a more refined extraction of the variables contributing to age-related variance (Descriptive statistics on Tables S2 and S3). The high Q^2^ values and significant CV-ANOVA *p* values confirm that the metabolic divergence between *P. sidoides* harvest ages is statistically robust and not merely a result of supervised model over-fitting.

Volcano plots were created using OPLS-DA descriptive statistics to visualize statistically significant and changes in metabolite abundance amongst harvest ages. The plots were created using the VolcaNoseR tool (https://huygens.science.uva.nl/VolcaNoseR/) with a significance threshold of − 10 log(p) > 1.3 and ± 0.3-fold change threshold. In the 6- versus 12-month harvest age comparisons, a disaccharide with RT/*m/z* 1.226/341.10965, tentatively identified as trehalose/sucrose, was increased (0.2431-fold) in the 6 months harvest age. This finding supports that young roots may prioritise primary metabolites for energy storage and stress protection, thus supporting root growth and development (Figueroa & Lunn, 2016; Göbel & Fichtner, 2023). In contrast, gallic acid (RT/*m/z* 3.024/169.0147) increased by 7.3175-foldnat 12 months harvest age suggestive of a transition to secondary metabolite biosynthesis that might contribute to enhanced antioxidant and anti-inflammatory activity in senescent plants (Kahkeshani et al., 2019). These patterns reflect a developmental transition from growth largely supported by primary metabolism in young plants to a prioritization of secondary metabolism in older plants (Jandova & Dolezal, 2025).

**Fig. 4:**
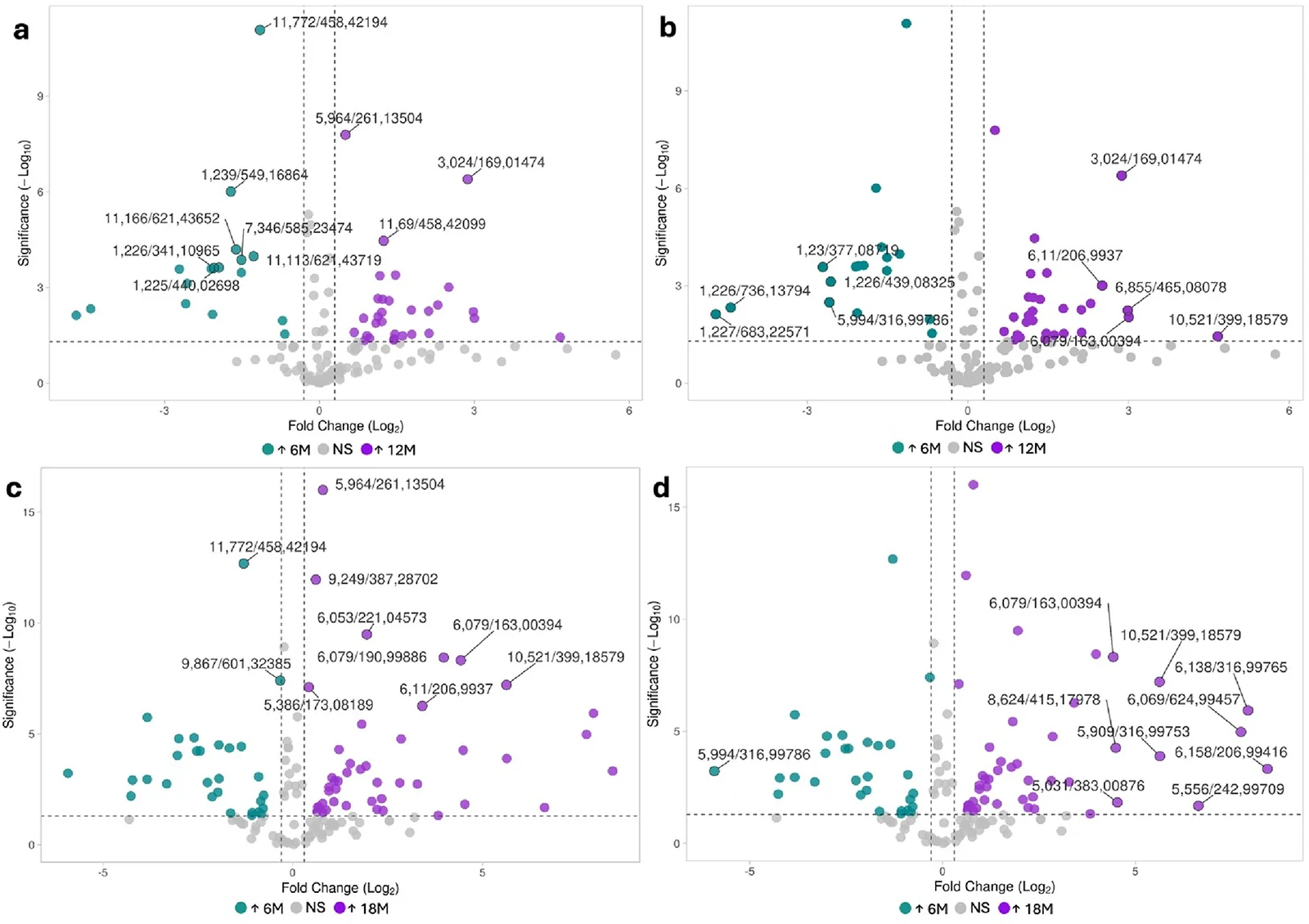
Volcano plots comparing metabolites between harvest ages. **a**, **c** highlight the top 10 statistically significant metabolites (based on *p*value), labelled by retention time/*m/z*, for the 6-month versus 12-month and 6-month versus 18-month comparisons, respectively. **b**, **d** display the top 10 metabolites with the largest fold changes for the same comparisons. Abbreviations; ↑ 6 M, increased in 6 months; ↑ 12 M, increased in 12 months; ↑ 18 M, increased in 18 months; *NS* not significant

Comparing 6- and 18-months harvest ages showed distinctive variations in their metabolic profiles, with a dominance of most significantly affected metabolites in 18-month samples. Isofraxidin/fraxinol (RT 6.053 min; *m/z* 221.04573) was identified as a major metabolite that was increased (3.8627-fold) at 18 months. In addition, two unknown sulphated flavonoids reported by Brendler et al. (2024), identified at RT 10.521 min (*m/z* 399.18579) and RT 8.624 min (*m/z* 415.17978), were also noted among the most significantly increased compounds, suggesting a developmental shift towards secondary metabolites accumulation. In contrast, an unknown compound detected at RT 5.994 min (*m/z* 316.99786) was abundant in 6-month samples, but its relative abundance was reduced with progressing samples harvest age. Two features with identical nominal mass but different retention time with this compound (RT 6.138 min; *m/z* 316.99765 and RT 5.909 min; *m/z* 316.99753) increased in the 18-month harvest. These features share mass with 8-hydroxy-5,7-dimethoxycoumarin-6-sulphate, previously identified in *P. sidoides* roots by Panara et al. (2022), suggesting a possible structural relationship between the compounds. These findings suggest that related compounds may be regulated differently as the plant grows, with certain isomers becoming more prominent at specific harvest ages (Fig. 4).

**Fig. 5:**
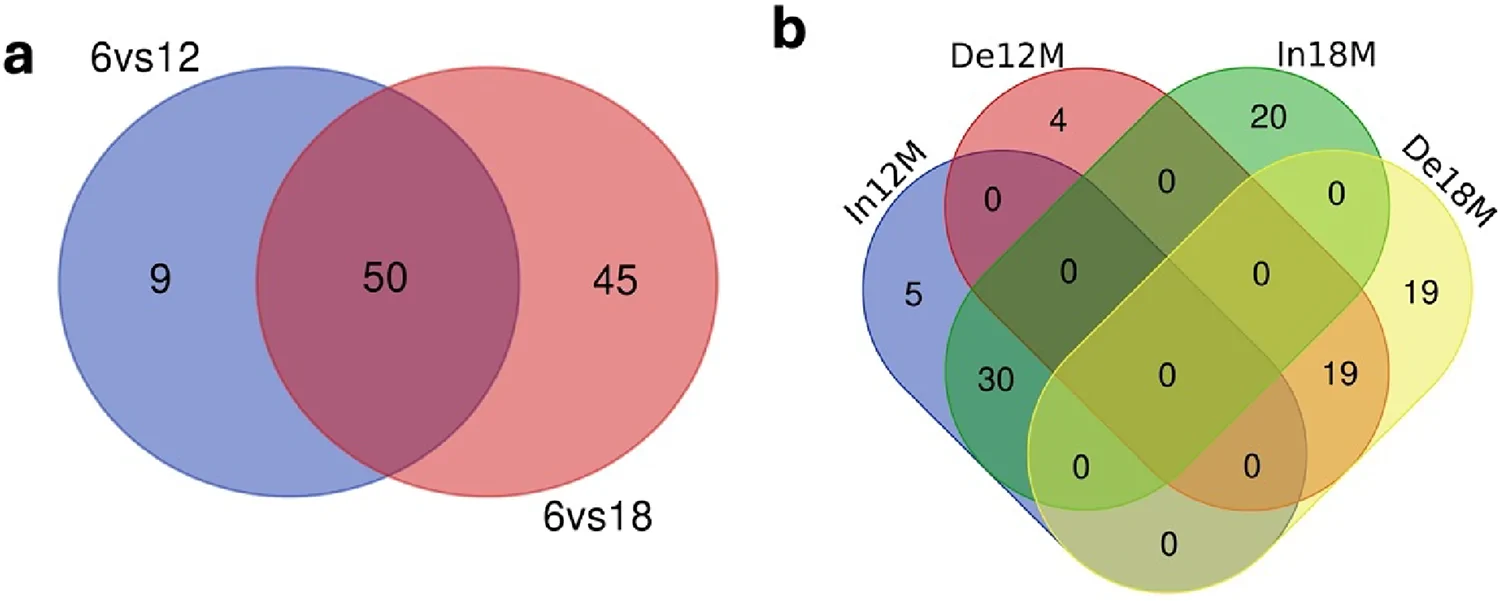
Comparative profiles of significant metabolites **a** between the OPLS-DA models for 6 months versus 12 months (6 vs 12) and 6 months versus 18 months (6 vs 18). **b** further shows features that were increased (In) or decreased (De) in the 12-month (12 M) and 18-month (18 M) harvest age. *In12M* Increased in 12 months, *De12M* decreased in 12 months, *In18M* increased in 18 months, *De18M* decreased in 18 months

To further evaluate the significant features from the OPLS-DA model, Venn diagrams (https://bioinformatics.psb.ugent.be/webtools/Venn/) were used (features on Tables S5 and S6). Comparisons between 6- versus 12-month harvest ages and 6- versus 18-month samples ages (Fig. 5a) showed significant overlap, with 50 of the features being significant in both comparisons, indicating a continuous change from 6 to 18 months harvest ages. Nine (9) of the features including dihydroxy coumarin sulphate were only significantly affected when comparing the 6- and 12-months harvest ages. While 45 features including scopoletin sulphate, umckalin sulphate, and epigallocatechin were significantly affected across 6 versus 18 months harvest ages which indicated further changes in metabolism at the later harvesting age. A four-way Venn diagram (Fig. 5b) further illustrates the dynamics of metabolite regulation, 35 metabolites were increased at 12 months, and 30 of these remained increased again at 18 months, suggesting stable regulation in the synthesis of compounds such as isofraxidin/fraxinol, umckalin epigallocatechin dimer and kuaburaside as the plant ages.

#### False discovery rate (FDR) correction using the Benjamini–Hochberg method

**Fig. 6:**
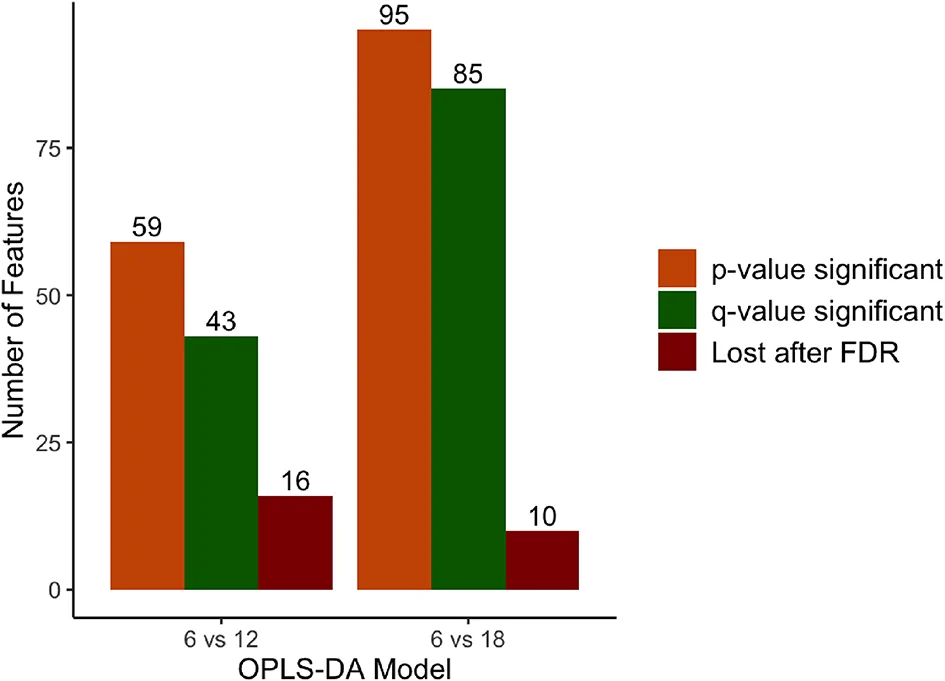
Summary of statistically significant features before and after Benjamini–Hochberg FDR correction, raw *p* value significance (*p* < 0.05), retained q-value significance (q < 0.05), and features lost after FDR adjustment

To mitigate the risk of false-positive findings inherent in multiple hypothesis testing, False Discovery Rate (FDR) correction was applied to the p-values derived from the OPLS-DA models using the Benjamini–Hochberg procedure (Tables S2 and S3). This approach controlled for Type I error and enhanced the robustness of the discriminatory features identified between sample groups (Peluso et al., 2021). Figure 6 shows how significant features were affected after FDR correction. In the comparison between 6-month and 12-month samples, 59 features were initially significant (*p* < 0.05); after FDR correction, 43 features retained significance (q < 0.05), while 16 features lost statistical significance. The observation that a substantial proportion (73%) of these features remained robust after adjustment supports the reliability of the metabolic separation between these developmental stages. Similarly, in the 6-month versus 18-month comparison, 95 features were initially significant, with 85 (89.5%) retaining significance after FDR adjustment. This indicates a profound and consistent metabolic shift between early and late harvest stages of *P. sidoides*, with minimal inflation of false positives among the model-derived features.

Considering identified metabolites, compounds such as umckalin, isofraxidin/fraxinol, gallic acid, and koaburaside remained significant after FDR correction, with umckalin, a known marker compound consistently discriminating between harvest stages. However, some metabolites, including epigallocatechin dimer (6 vs. 12 months), scopoletin sulphate, and trehalose/sucrose (6 vs. 18 months), did not retain significance after adjustment, indicating that their initial significance was likely influenced by multiple comparisons and may not represent truly robust discriminatory features.

#### Annotated metabolites change across harvest ages

Analysis of the significant changes of annotated metabolites is presented in Table 2, in the 6- versus 12-months harvest ages comparison significant increase of compounds such as dihydroxy coumarin sulphate, isofraxidin, 6,8-dihydroxy-5,7-dimethoxy coumarin, umckalin, epigallocatechin dimer and gallic acid with fold changes ranging from 1.6 to 7.3. In contrast, 6-month harvest age exhibited higher levels of trehalose/sucrose with 0.24-fold change. This pattern suggests a developmental shift in metabolic allocation, younger *P. sidoides* roots accumulated sugars, whereas older roots allocated more resources toward the synthesis of phenolic compounds and coumarin derivatives (Li et al., 2020).

In the 6- versus 18-months harvest age comparison, coumarins such as isofraxidin/fraxinol and 6,8-dihydroxy-5,7-dimethoxy coumarin, and flavan-3-ols including epigallocatechin, gallocatechin dimers and trimers, were increased (1.5–3.8-fold change), reflecting increased secondary metabolite biosynthesis in older plants (Table 2). Gallic acid and koaburaside also exhibited an increase with fold changes 4.2 and 3.8, respectively. This pattern is consistent with the general trend in perennial plants, where phenolic compounds increase with age (Hazrati et al., 2024). Compounds such as Trehalose/sucrose, scopoletin sulphate, 8-hydroxy-5,7-dimethoxycoumarin-6-sulphate, umckalin sulphate, and galloyl-apigenin-C-hexoside decreased at 18 months harvest age (fold changes 0.12–0.58), indicating selective metabolic changes as the plant grows. Although the regulation of sulphated secondary metabolites in plants is not well established, the decline in sulphated metabolites in *P. sidoides* with age may possibly reflect ontogenetic shifts in metabolism, involving enzymatic de-sulphation or reallocation of sulphur, leading to reduced storage conjugates and increased accumulation of free bioactive compounds (Kopriva et al., 2024; Takahashi et al., 2023).

Other studies have also reported the age-dependent changes in secondary metabolites across medicinal plants. For example, Zhao et al. (2023) examined *Sinopodophyllum hexandrum* between 2 and 9 years and reported that flavonoids and phenolic compounds peaked at 5–6 years, with the expression of eight key genes involved in lignan and flavonoid biosynthesis being up-regulated as the plants aged. Similarly, in *Stellaria dichotoma* L. var. *lanceolata Bge* (SDL), different metabolite profiles were observed at different ages, 1–2-year-old plants favoured lipid accumulation, whereas 3–5-year-old plants contained more alkaloids and benzenoids. The study reported an increase of 12 metabolites with age and a decline in about 20 metabolites, resulting in 3-year-old SDL showing the optimum harvest time for SDL plant (Li et al., 2023). Li et al. (2020) investigated *Cinnamomum loureirii* bark from trees aged 6–15 years and identified 13–14 years as the optimal harvest age, as the highest phenolic, flavonoid, and antioxidant activity were obtained at this harvest age. Trees aged 10–12 years were reported to accumulate more trans-cinnamaldehyde, and older trees (13–15 years) contained higher levels of α-copaene. These studies reflect that plant harvest age affect the type and concentration of secondary metabolites, with optimum years of harvesting varying with species depending on biosynthetic processes and classes of metabolites of interest.

**Table 2:** Significant metabolites contributing to differences across 6-, 12-, and 18-month harvests

Compound name		RT (minutes)	m/z	*p* value	q-value	Fold change
6 months versus 12 months						
Dihydroxy coumarin sulphate		3.963	272.9714	0.01315	0.04372	2.13549
Isofraxidin/fraxinol		6.053	221.0457	0.00917	0.03278	1.81131
6.8-Dihydroxy-5.7-dimethoxy coumarin		6.162	237.0406	0.00040	0.00320	2.77940
Umckalin		6.847	221.0460	0.00042	0.00320	2.25609
Epigallocatechin dimer		3.296	609.1270	0.02550	0.07596	1.59636
Galloyl-apigenin-C-hexoside		7.166	585.2344	0.000342	0.00288	0.351204
Gallic acid		3.024	169.0142	4.00937 × 10^− 07^	1.91 × 10^− 05^	7.31748
Koaburaside		3.094	331.1037	0.00221	0.01264	2.19467
Trehalose/sucrose		1.226	341.1097	0.00024	0.00236	0.24312
6 months versus 18 months						
Scopoletin sulphate		4.910	270.9924	0.03566	0.05682	0.47776
8 Hydroxy 5.7 dimethoxycoumarin-6-sulphate		5.813	316.9972	9.35548 × 10^− 5^	0.00045	0.12185
Isofraxidin/fraxinol		6.053	221.0457	3.25285 × 10^− 10^	1.18 × 10^− 08^	3.86270
Umckalin sulphate		6.001	301.0024	3.11259 × 10^− 5^	0.00022	0.25977
6.8-Dihydroxy-5.7-dimethoxy coumarin		6.162	237.0406	0.01153	0.02290	2.12124
Umckalin		6.847	221.0460	0.01362	0.02670	1.72778
(−)-Epigallocatechin		3.947	305.0674	0.00096	0.00316	2.05038
Epigallocatechin dimer		3.296	609.1270	0.00254	0.00624	1.93885
Gallocatechin dimer		3.567	609.1253	0.03456	0.05569	1.73352
Epigallocatechin trimer		3.189	913.1855	0.03225	0.05375	1.56365
Gallocatechin trimer		3.383	913.1838	0.03146	0.05304	1.56754
Galloyl-apigenin-C-hexoside		7.166	585.2343	0.02277	0.04076	0.58262
Gallic acid		3.024	169.0142	0.01102	0.02220	4.23578
Koaburaside		3.094	331.1037	0.00028	0.00113	3.81074
Trehalose/sucrose		1.226	341.1097	0.04185	0.06525	0.56005

*RT* Retention time, *m/z* mass to charge ratio

### Targeted metabolite semi-quantification

**Fig. 7:**
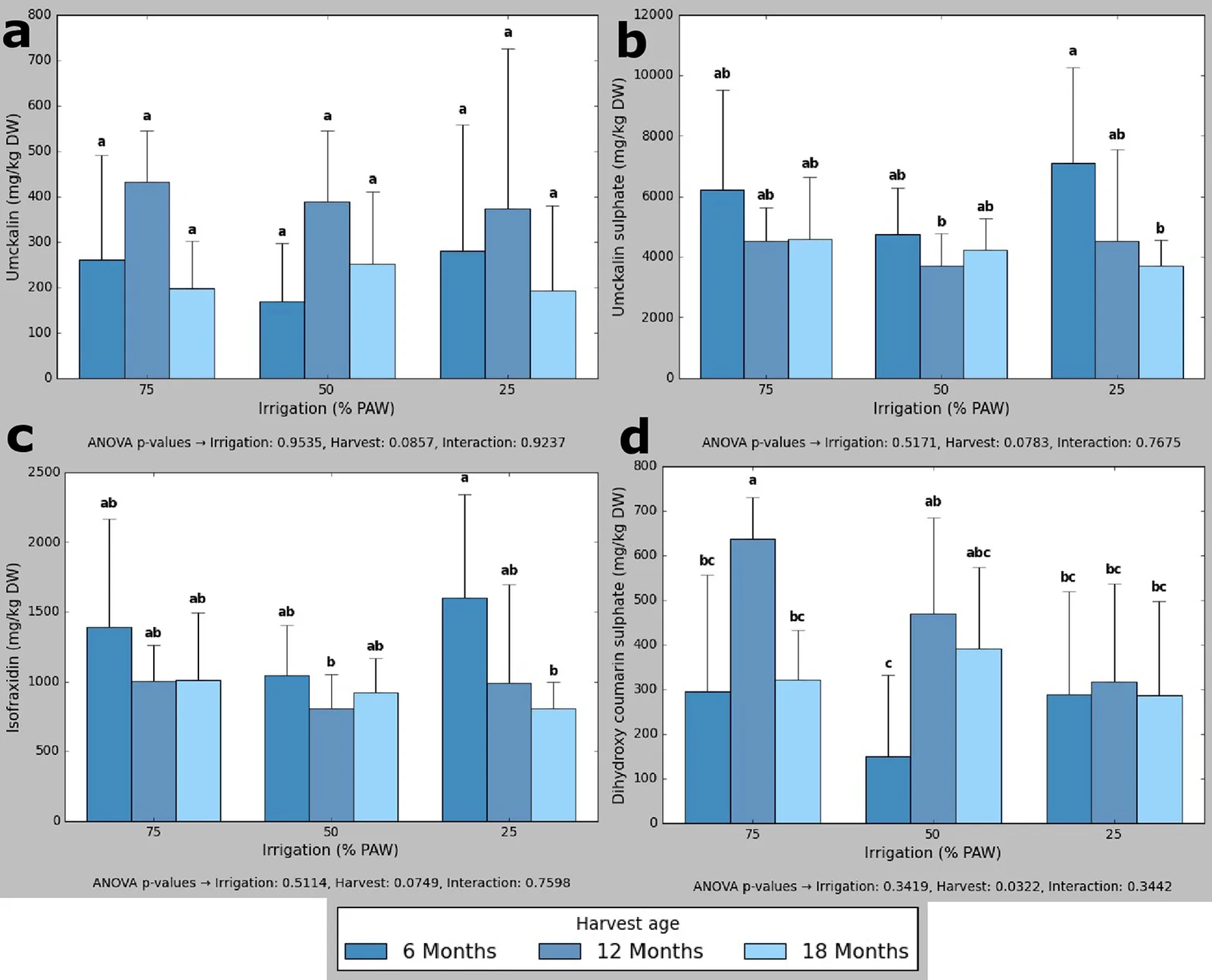
Quantified coumarins; umckalin (**a**), umckalin sulphate (**b**), isofraxidin (**c**), and dihydroxycoumarin sulphate (**d**) in *P. sidoides* roots across different harvest ages and irrigation levels (plant available water, PAW). Distinct letters indicate significant differences between samples (*P* < 0.05)

**Fig. 8:**
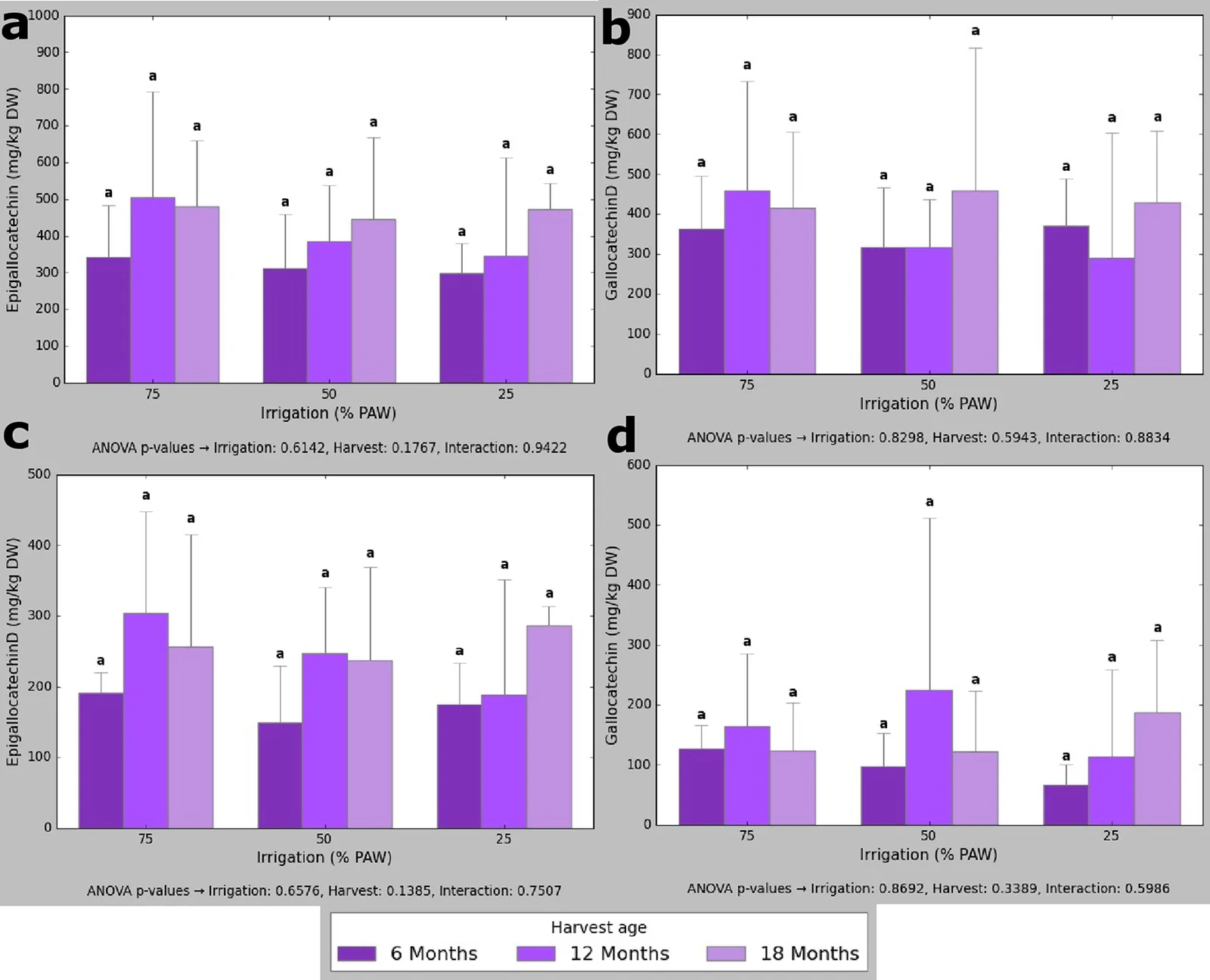
Quantified flavan-3-ols, epigallocatechin (**a**), gallocatechin (**b**), epigallocatechin dimer (EpigallocatechinD, **c**), and gallocatechin dimer (GallocatechinD, **d**) in *Pelargonium sidoides* roots across different harvest ages and irrigation levels (25%, 50%, and 75% PAW). Distinct letters indicate significant differences between samples (*P* < 0.05)

The quantification of metabolites in *P. sidoides* was done in two groups, umckalin related (Fig. 7) and epigallocatechin related (Fig. 8) compounds. Although no statistical significance was observed on the umckalin different treatment groups an interesting trend was observed. The highest amount was observed at 75% PAW, 12 months (431.61 ± 112.94 mg/kg), with the lowest values observed at 50% PAW 6 months harvest age (169.28 ± 127.73). The umckalin concentrations observed in this are comparable to cultivation of *P. sidoides* study with application of irrigation and nitrogen done by Mofokeng et al. (2020), they study reported 38.3 to 45.3 mg/100g^− 1^ (383 to 453 mg/kg). However, a study that quantified umckalin in wild cultivated plant of unknown age cultivated at different locations reported between 3741 and 26,249 mg/kg umckalin extracted using 11% and 60% ethanol (Van Wyngaard et al., 2023). This suggest that umckalin may be influenced by factors such as plant age, genotype and environmental conditions.

The sulphated form of umckalin, was highest at 25% PAW, at 6 months harvest age (7110.32 ± 3136.37), with significantly lower concentrations observed at 50% PAW 12 months harvest age (3715.15 ± 1070.84), and 25% 18 months harvest age (3720.55 ± 828.29). The high amount of umckalin sulphate at 6 months harvest age may be due to developmental metabolic activity, at which sulphation increases solubility, facilitate transport and storage of metabolites (Han et al., 2025). Dihydroxy coumarin sulphate and isofraxidin/fraxinol were quantified also, although not statistically significant lowest values were observed at the lowest irrigation (25%) at both 12- and 18-months harvest age. Dihydroxy coumarin sulphate peaked at 75% PAW, 12 months harvest age (636.44 ± 93.53), following a trend on umckalin levels, although other compounds are expressed as umckalin equivalents.

The quantification of flavo-3-ols (Fig. 8) showed no statistical difference as affected by irrigation, harvest age and interaction between harvest age and irrigation. The results however showed highest amount of epigallocatechin and gallocatechin dimer at 75% PAW, 12 months harvest. Gallocatechin was highest at 50% PAW, 12 months harvest age while its dimer was highest at 50% PAW 18 months, these results shows an intricate mechanism of production of these metabolites between isomers and dimers of these compounds, for instance the epi isomers shows higher concentration than its dimers, however the gallocatechin shows less than its dimer, this suggests that gallocatechin may easily form dimers than the epigallocatechin isomer. Gallocatechin dimer is also higher at 18 months harvest age. In addition, across these compounds the most abundant was epigallocatechin, similar results were obtained by Savickiene et al. (2018) where epigallocatechin was significantly higher compared to other compounds such as epicatechin and catechin.

## Conclusions

This study evaluated the effect of irrigation and harvest age on the metabolites of *P. sidoides* roots. Irrigation did not result in statistically significant changes in metabolite profiles across the harvest ages studied, suggesting limited influence under the irrigation levels studied. The observed differences were significant and more pronounced for harvest age effects. Multivariate OPLS-DA showed that harvest age affected the concentration of metabolites obtained, high concentrations of secondary metabolites were observed in the 18 months harvest age. It is important to note that some modified secondary metabolites were higher at 6 months, which suggests the intricate metabolism of secondary metabolites in *P. sidoides* roots. Furthermore, the sustainable cultivation of *P. sidoides* is feasible at wide range of water availability as the plant maintains its metabolite profile. This study also highlights that season may be of important when considering the optimal harvest of *P. sidoides*. Compounds such as gallic acid, 6.8-dihydroxy-5.7-dimethoxy coumarin, and umckalin were highest at 12 months age which was harvested during the winter season. It also indicated an ontogenetic increase of metabolites, with 18 months harvest containing more significantly increased metabolites such as isofraxidin/fraxinol, epigallocatechin dimer, and kouburaside. An optimal harvest was not determined; thus longer-term studies are needed to determine the optimum age of harvest and long-term effects of irrigation regimes.

## Supplementary Information

Below is the link to the electronic supplementary material.

Supplementary Material 1

## Data Availability

Processed MS feature data (m/z, RT, MS/MS fragments) are included in the Supplementary Information.
