# Supplementary material for: Ultra-performance liquid chromatography-quadrupole time-of-flight mass spectrometry metabolomic profiling reveals harvest age dependent changes in the roots of Pelargonium sidoides DC
Source: Metabolomics. 2026 Jul 8;22(4):120. doi: 10.1007/s11306-026-02505-6 (PMC13346132; doi:10.1007/s11306-026-02505-6)
Supplement: Supplementary file 1 — Supplementary Material 1 [file 11306_2026_2505_MOESM1_ESM.docx]

**UPLC-QTOF-MS metabolomic profiling reveals harvest age dependent changes in the roots of *Pelargonium sidoides* DC.**

Kundani Khameli^1,2^ <https://orcid.org/0000-0002-4203-0203>,

Muinat Nike Lewu^3^, <https://orcid.org/0000-0002-8945-8100> ,

Takalani Mulaudzi^4^ <https://orcid.org/0000-0002-5792-1973> ,

Oluwafemi Ayodeji Adebo^1^*<https://orcid.org/0000-0002-3757-5137>

Samuel Oluwatobi Oluwafemi^2^ <https://orcid.org/0000-0001-8964-5646> ,

^1^Centre for Innovative Food Research (CIFR), Department of Biotechnology and Food Technology, Faculty of Science, University of Johannesburg, Doornfontein, P.O. Box 17011, Johannesburg 2028, Gauteng, South Africa.

^2^Centre for Nanomaterials Science Research, Department of Chemical Sciences, University of Johannesburg, Doornfontein Campus, P.O. Box 17011, Doornfontein 2028, South Africa.

^3^Soil and Water Science Programme, Agricultural Research Council Infruitec-Nietvoorbij, Private Bag X5026, Stellenbosch 7599, South Africa.

^4^Life Sciences Building, Department of Biotechnology, University of the Western Cape, Private Bag X17, Bellville 7535, South Africa.

Corresponding author: *[oadebo@uj.ac.za](mailto:oadebo@uj.ac.za), [oaadebo@gmail.com](mailto:oaadebo@gmail.com)

**SUPPLEMENTARY**

**
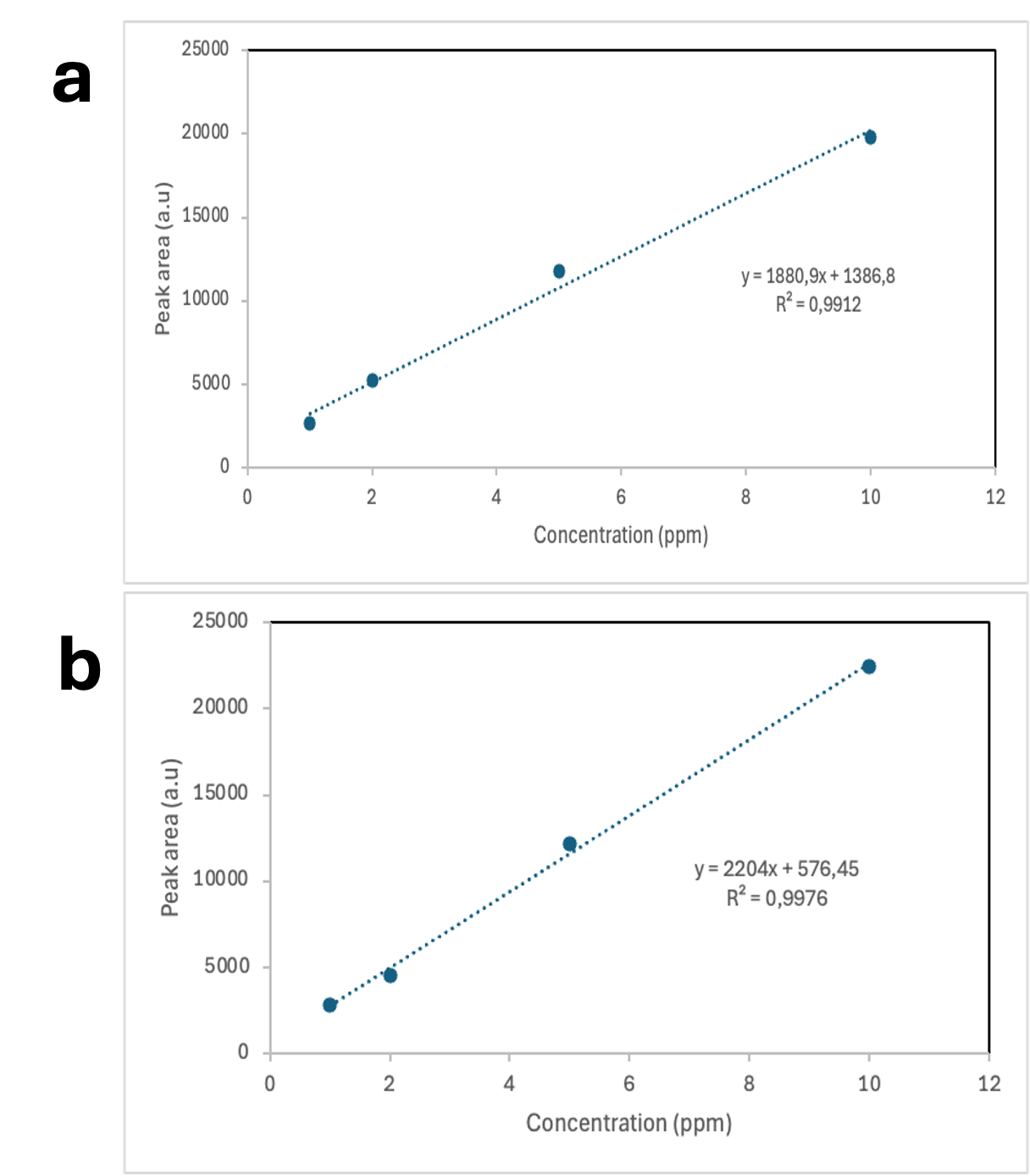
**

**Fig. S1** Umckalin (a) and Epigallocatechin (b) standard curves used for semi-quantification of related compounds in *P. sidoides* root samples.


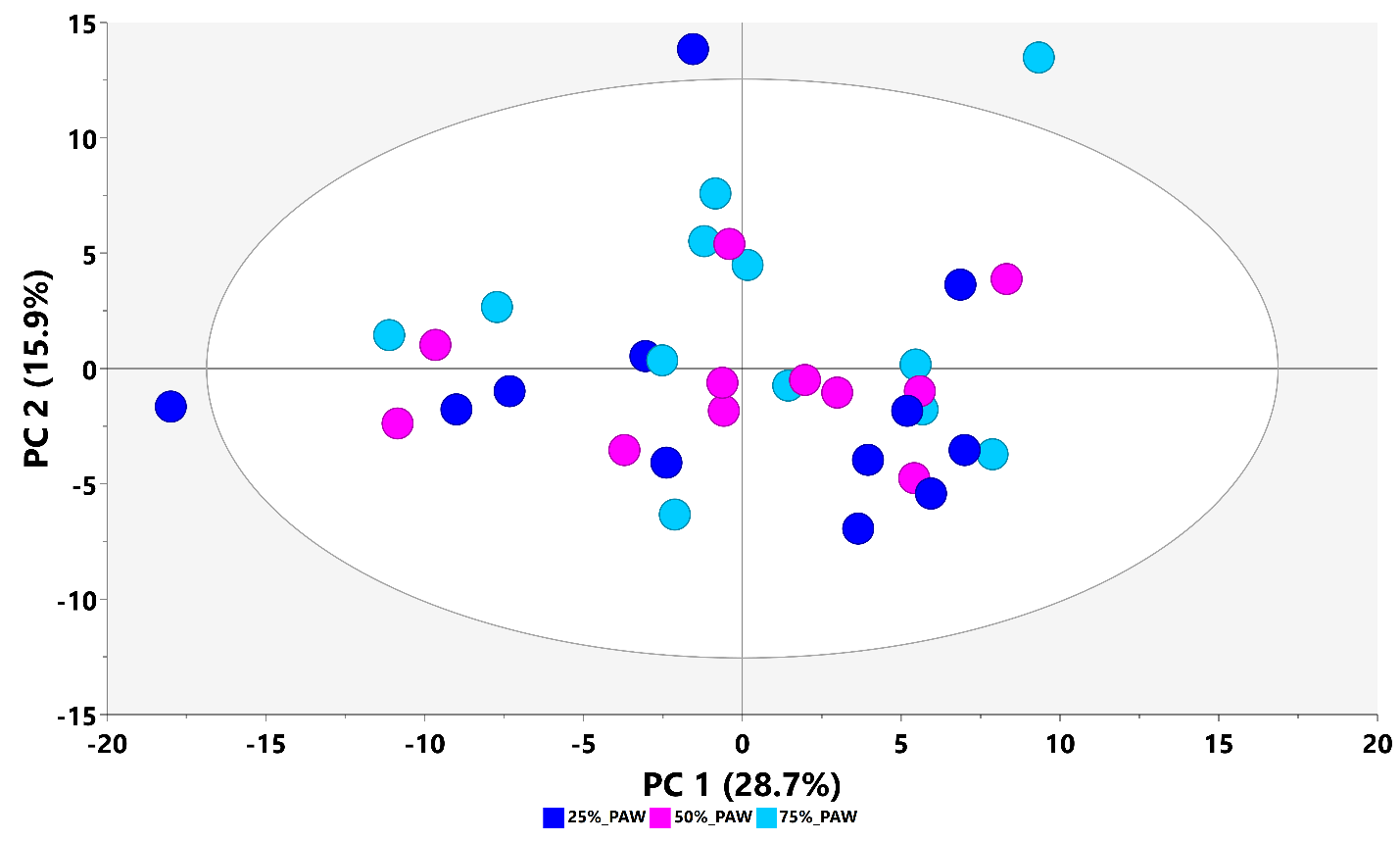


**Fig. S2** Principal component analysis scores visualised by irrigation at 25, 50 and 75% plant available water (PAW).


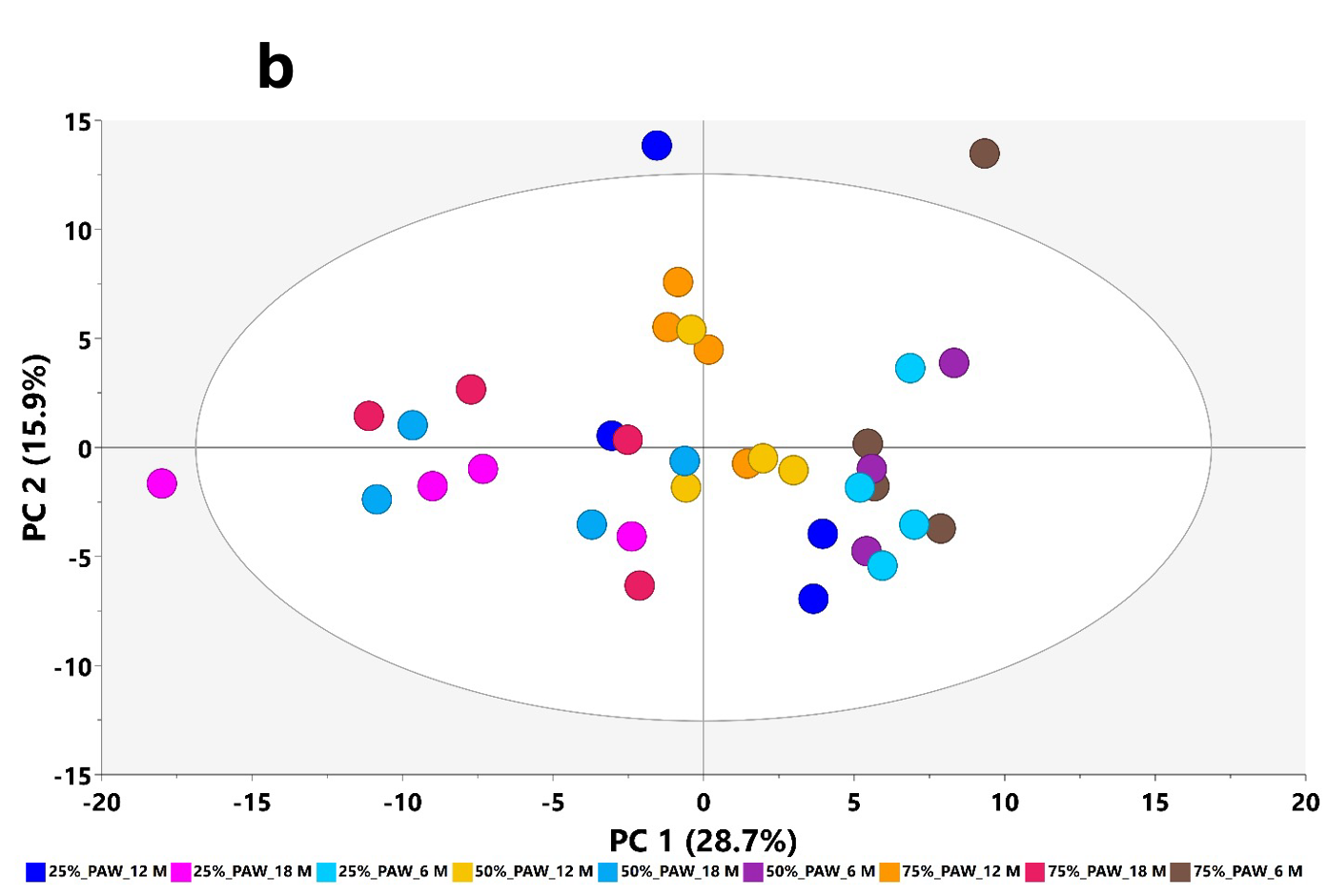


**Fig. S3** Principal component analysis scores visualised by interaction of irrigation (25, 50 and 75% PAW) and harvest age (6, 12, and 18 months).

**Table S1** Metabolite features obtained from samples of *P. sidoides* root extract on UPLC-QTOF-MS.

| **Average Rt (min)** | ***m/z*** | **MS/MS** Fragments | **Identification Status** |
| --- | --- | --- | --- |
| 1,224 | 387,1153 | 341.10919, 342.11392, 377.08609, 383.12051, 387.11438 | Unidentified |
| 1,225 | 440,027 | 341.10919, 342.11392, 377.08609, 383.12051, 387.11438, 404.10529, 440.02997 | Unidentified |
| 1,226 | 341,1097 | 341.10919 | Identified |
| 1,226 | 439,0833 | 341.10931, 342.11292, 377.08646, 379.08298, 383.12085, 387.11493, 404.10608, 429.996 | Unidentified |
| 1,226 | 736,1379 | 341.10974, 342.11316, 377.08704, 379.0853, 387.11469, 395.0289, 404.1058, 440.02701, 441.03302, 454.04211, 508.96921, 556.07373, 574.08112, 683.22485, 684.22931, 703.2326, 736.13629 | Unidentified |
| 1,227 | 683,2257 | 341.10974, 342.11316, 377.08704, 379.0853, 387.11469, 395.0289, 404.1058, 440.02701, 441.03302, 454.04211, 508.96921, 556.07373, 574.08112, 683.22485 | Unidentified |
| 1,23 | 377,0872 | 341.10931, 342.11292, 377.08646 | Unidentified |
| 1,236 | 711,2219 | 341.10925, 377.08539, 383.11859, 387.11594, 404.10339, 503.16211, 549.16718, 665.21362, 711.22125 | Unidentified |
| 1,239 | 549,1686 | 341.11035, 383.11957, 387.11591, 404.10437, 503.1611, 549.16528 | Unidentified |
| 2,004 | 554,262 | 145.93092, 146.93845, 174.95593, 190.92921, 206.97289, 235.92592, 239.90356, 263.86554, 307.85519, 352.85489 | Unidentified |
| 2,887 | 216,0337 | 208.09807, 216.03381 | Unidentified |
| 2,911 | 328,046 | 145.93221, 146.93855, 174.95558, 206.97301, 235.92667, 282.08481, 328.04602 | Identified |
| 2,963 | 344,0403 | 305.01797, 306.02176, 326.01346, 331.06644, 344.04056 | Identified |
| 3,022 | 391,9489 | 169.01477, 303.96829, 347.01993, 347.96002, 362.99643, 391.94861 | Unidentified |
| 3,024 | 169,0147 | 169.01477 | Identified |
| 3,026 | 125,0248 | ND | Unidentified |
| 3,028 | 609,1255 | 305.06628, 423.0737, 609.12524 | Unidentified |
| 3,094 | 331,1037 | ND | Identified |
| 3,16 | 358,0568 | 358.05508 | Unidentified |
| 3,189 | 913,1855 | 305.06567, 423.073, 541.07617, 913.18152 | Identified |
| 3,281 | 411,0246 | 305.06662, 411.02481 | Unidentified |
| 3,29 | 609,1259 | 305.06558, 423.07135, 609.1225 | Identified |
| 3,353 | 272,9712 | ND | Unidentified |
| 3,383 | 913,1838 | 305.06631, 423.07275, 727.12994, 913.1825 | Identified |
| 3,429 | 272,9716 | ND | Unidentified |
| 3,476 | 305,0669 | 261.04227, 303.05075, 305.06717 | Identified |
| 3,477 | 913,1859 | 243.03108, 261.04153, 303.05014, 305.06726, 306.06952, 423.07321, 424.077, 441.08511, 481.07712, 483.09433, 541.07867, 559.08716, 607.11487, 608.11829, 609.12476, 610.12622, 611.1366, 727.1344, 745.1394, 760.14899, 760.64612, 911.16718, 912.1701, 913.18335 | Unidentified |
| 3,512 | 760,1456 | 177.019, 243.0304, 261.04074, 303.05157, 305.06686, 421.05679, 423.0733, 424.07803, 429.08432, 439.06677, 441.08441, 481.07669, 541.07446, 607.11029, 608.11774, 609.12341, 727.13116, 760.14294 | Unidentified |
| 3,527 | 608,1174 | 177.01866, 261.04059, 303.05139, 305.06775, 423.07297, 481.07843, 607.10785, 608.11578 | Unidentified |
| 3,54 | 913,1818 | 177.01942, 243.03032, 255.02943, 261.04053, 273.04086, 283.02603, 297.04135, 303.05289, 305.06717, 306.07117, 423.07306, 424.07758, 439.0694, 441.08224, 481.07727, 483.09021, 541.0769, 607.10962, 608.11621, 609.12531, 610.1275, 611.13428, 727.1308, 911.16547, 912.17737, 913.18317 | Unidentified |
| 3,559 | 609,1262 | 177.02155, 305.06702, 423.07303, 424.07681, 607.1095, 609.12543 | Identified |
| 3,618 | 593,132 | 177.01974, 261.04175, 303.05035, 305.06729, 423.07288, 439.06491, 593.12738 | Unidentified |
| 3,947 | 305,0674 | 193.01353, 272.97165, 305.06644 | Identified |
| 3,948 | 404,9918 | 193.01472, 272.97189, 273.97449, 303.05151, 305.06631, 306.07083, 358.98584, 365.01968, 368.06067 | Unidentified |
| 3,948 | 593,1315 | 303.05313, 305.06741, 306.07153, 423.07431, 481.07977, 593.13428 | Unidentified |
| 3,953 | 611,1417 | 193.01376, 272.97098, 273.97314, 303.0517, 305.06638, 306.06985, 358.98657, 365.01993, 368.06152, 607.10889, 611.14075 | Unidentified |
| 3,958 | 633,1235 | 165.0193, 167.035, 179.03555, 219.06749, 221.04568, 221.96175, 261.07755, 303.05215, 305.06705, 306.07068, 307.07291, 327.04919, 330.99033, 331.99881, 341.04379, 343.02652, 358.98676, 359.99374, 368.06265, 387.98987, 526.02191, 527.02563, 609.13159, 611.14148, 612.14484, 613.14709, 633.12579 | Unidentified |
| 3,958 | 664,0524 | 165.0193, 167.035, 179.03555, 219.06749, 221.04568, 221.96175, 261.07755, 303.05215, 305.06705, 306.07068, 307.07291, 327.04919, 330.99033, 331.99881, 341.04379, 343.02652, 358.98676, 359.99374, 368.06265, 387.98987, 526.02191, 527.02563, 609.13159, 611.14148, 612.14484, 613.14709, 633.12579, 649.09961, 664.05292 | Unidentified |
| 3,959 | 193,0146 | 193.01424 | Unidentified |
| 3,961 | 373,0549 | 165.0193, 167.035, 179.03555, 219.06749, 221.04568, 221.96175, 261.07755, 303.05215, 305.06705, 306.07068, 307.07291, 327.04919, 330.99033, 331.99881, 341.04379, 343.02652, 358.98676, 359.99374, 368.06265 | Unidentified |
| 3,961 | 449,9913 | 165.0193, 167.035, 179.03555, 219.06749, 221.04568, 221.96175, 261.07755, 303.05215, 305.06705, 306.07068, 307.07291, 327.04919, 330.99033, 331.99881, 341.04379, 343.02652, 358.98676, 359.99374, 368.06265, 387.98987 | Unidentified |
| 3,962 | 303,0512 | 165.01964, 167.03574, 179.03442, 219.06647, 221.04668, 261.07748, 303.05136 | Unidentified |
| 3,963 | 272,9714 | 193.01424, 272.97156 | Identified |
| 3,964 | 365,0194 | 305.06763 | Unidentified |
| 4,006 | 554,262 | 145.93109, 146.93857, 174.95656, 193.01378, 206.97232, 235.92607, 239.90248, 272.97107, 273.97522, 274.97061, 294.95184, 305.06683, 359.10123 | Unidentified |
| 4,214 | 289,0722 | 203.07153, 221.08313, 245.08214, 246.08556, 287.05618, 288.06024, 289.07178, 289.25839 | Identified |
| 4,216 | 245,0822 | 203.07153, 221.08313, 245.08214 | Unidentified |
| 4,216 | 325,0499 | 203.07153, 221.08313, 245.08214, 246.08556, 287.05618, 288.06024, 289.07178, 289.25839, 290.0755, 291.07736, 292.08087, 303.05154, 307.0824, 311.05441, 312.05698, 325.04825 | Unidentified |
| 4,216 | 632,0638 | 203.07153, 221.08313, 245.08214, 246.08556, 287.05618, 288.06024, 289.07178, 289.25839, 290.0755, 291.07736, 292.08087, 303.05154, 307.0824, 311.05441, 312.05698, 325.04825, 326.05191, 327.03983, 341.08789, 341.11111, 347.03226, 352.06985, 357.05942, 358.06262, 359.06448, 359.09906, 373.03146, 374.04187, 379.10553, 387.04797, 387.9921, 388.99667, 390.00171, 390.99796, 395.98944, 401.08597, 407.99429, 419.02866, 425.04733, 432.00751, 433.99426, 434.99796, 480.00131, 481.00198, 483.97849, 577.13574, 578.1391, 579.15137, 580.15497, 581.15839, 582.16272, 597.15857, 601.13281, 602.14154, 603.16833, 615.12646, 617.10748, 618.11285, 619.11267, 630.07001, 631.1875, 632.06189 | Unidentified |
| 4,219 | 579,1517 | 203.07153, 221.08313, 245.08214, 246.08556, 287.05618, 288.06024, 289.07178, 289.25839, 290.0755, 291.07736, 292.08087, 303.05154, 307.0824, 311.05441, 312.05698, 325.04825, 326.05191, 327.03983, 341.08789, 341.11111, 347.03226, 352.06985, 357.05942, 358.06262, 359.06448, 359.09906, 373.03146, 374.04187, 379.10553, 387.04797, 387.9921, 388.99667, 390.00171, 390.99796, 395.98944, 401.08597, 407.99429, 419.02866, 425.04733, 432.00751, 433.99426, 434.99796, 480.00131, 481.00198, 483.97849, 577.13574, 578.1391, 579.15137 | Unidentified |
| 4,547 | 369,0834 | 369.08304 | Identified |
| 4,623 | 457,0788 | 169.01457, 287.05621, 289.07196, 305.06714, 427.06635, 453.04797, 454.05063, 455.06302, 456.06683, 457.03833, 457.07874, 457.31494 | Unidentified |
| 4,627 | 169,0148 | 169.01457 | Unidentified |
| 4,627 | 520,0747 | 169.01457, 287.05621, 289.07196, 305.06714, 427.06635, 453.04797, 454.05063, 455.06302, 456.06683, 457.03833, 457.07874, 457.31494, 458.04166, 458.08215, 459.08459, 460.08795, 461.08868, 463.08868, 469.04059, 471.05649, 472.06125, 473.07321, 474.07437, 477.04501, 477.08478, 479.06097, 480.06512, 481.06769, 493.05579, 494.05896, 494.52609, 495.03995, 496.04291, 497.03894, 499.06531, 515.03809, 517.99365, 519, 519.99237, 520.07483 | Unidentified |
| 4,627 | 915,165 | 169.01457, 287.05621, 289.07196, 305.06714, 427.06635, 453.04797, 454.05063, 455.06302, 456.06683, 457.03833, 457.07874, 457.31494, 458.04166, 458.08215, 459.08459, 460.08795, 461.08868, 463.08868, 469.04059, 471.05649, 472.06125, 473.07321, 474.07437, 477.04501, 477.08478, 479.06097, 480.06512, 481.06769, 493.05579, 494.05896, 494.52609, 495.03995, 496.04291, 497.03894, 499.06531, 515.03809, 517.99365, 519, 519.99237, 520.07483, 521.07788, 522.08191, 525.06433, 526.06287, 529.99591, 530.99963, 541.03296, 542.04858, 545.96527, 547.04785, 552.00317, 553.9975, 554.98993, 555.05573, 555.99493, 556.05823, 557.00092, 558.00287, 559.00165, 561.00256, 563.02002, 569.09015, 571.07355, 572.07361, 575.01575, 576.02069, 577.0047, 577.03204, 577.97864, 578.98468, 586.12207, 600.0235, 605.97382, 609.02197, 722.55884, 723.06403, 723.56726, 724.06934, 724.57019, 731.55426, 734.56024, 747.16156, 911.12671, 913.15015, 914.15161, 915.16437 | Unidentified |
| 4,628 | 305,0674 | 169.01457, 287.05621, 289.07196, 305.06714 | Unidentified |
| 4,628 | 455,0638 | 169.01457, 287.05621, 289.07196, 305.06714, 427.06635, 453.04797, 454.05063, 455.06302 | Unidentified |
| 4,628 | 479,0612 | 169.01457, 287.05621, 289.07196, 305.06714, 427.06635, 453.04797, 454.05063, 455.06302, 456.06683, 457.03833, 457.07874, 457.31494, 458.04166, 458.08215, 459.08459, 460.08795, 461.08868, 463.08868, 469.04059, 471.05649, 472.06125, 473.07321, 474.07437, 477.04501, 477.08478, 479.06097 | Unidentified |
| 4,628 | 493,0562 | 169.01457, 287.05621, 289.07196, 305.06714, 427.06635, 453.04797, 454.05063, 455.06302, 456.06683, 457.03833, 457.07874, 457.31494, 458.04166, 458.08215, 459.08459, 460.08795, 461.08868, 463.08868, 469.04059, 471.05649, 472.06125, 473.07321, 474.07437, 477.04501, 477.08478, 479.06097, 480.06512, 481.06769, 493.05579 | Unidentified |
| 4,91 | 270,9924 | 145.93121, 174.95584, 191.03503, 206.97221, 235.92664, 270.99231 | Identified |
| 4,93 | 207,0304 | 192.00648, 207.02992 | Identified |
| 4,963 | 270,9923 | 145.93115, 174.95671, 191.03583, 235.92607, 270.99222 | Unidentified |
| 5,007 | 191,0353 | 191.03526 | identified |
| 5,018 | 270,992 | 191.0354, 270.99249 | Unidentified |
| 5,031 | 383,0088 | ND | Unidentified |
| 5,072 | 207,0303 | ND | Unidentified |
| 5,113 | 575,1629 | 190.99971, 221.04655, 575.16138 | Unidentified |
| 5,128 | 383,0082 | 383.00882 | Unidentified |
| 5,184 | 383,0082 | ND | Unidentified |
| 5,195 | 773,2521 | 459.14966, 773.25146 | Unidentified |
| 5,204 | 301,0024 | 301.0007 | Identified |
| 5,226 | 429,1051 | 429.10278 | Unidentified |
| 5,254 | 581,2239 | 581.22504 | Unidentified |
| 5,277 | 301,0032 | 301.00317 | Unidentified |
| 5,295 | 537,199 | 301.00464, 537.19897 | Unidentified |
| 5,295 | 583,2055 | 301.00464, 537.19897, 581.22461, 582.2298, 583.20691 | Unidentified |
| 5,316 | 206,0824 | ND | Unidentified |
| 5,317 | 441,0841 | ND | Unidentified |
| 5,372 | 581,189 | 343.11911, 551.21643, 581.18951 | Unidentified |
| 5,386 | 173,0819 | ND | Unidentified |
| 5,393 | 551,2136 | 551.21442 | Unidentified |
| 5,394 | 597,2202 | 343.11935, 389.16022, 551.21399, 552.21759, 581.18994, 597.2207 | Unidentified |
| 5,401 | 367,0132 | ND | Unidentified |
| 5,465 | 367,0138 | 367.01389 | Unidentified |
| 5,482 | 242,9974 | ND | Unidentified |
| 5,49 | 287,0564 | 259.0614,269.04529, 287.05661 | Identified |
| 5,493 | 301 | 299.9924, 300.99908 | Unidentified |
| 5,556 | 242,9971 | ND | Unidentified |
| 5,563 | 367,0131 | 367.01099 | Identified |
| 5,569 | 551,2131 | 371.11526, 373.13019, 419.17221, 551.21399 | Unidentified |
| 5,58 | 597,219 | 371.11526, 373.13019, 419.17221, 551.21399, 552.21741, 597.21887 | Unidentified |
| 5,617 | 787,2673 | 473.16641, 787.26898 | Unidentified |
| 5,633 | 397,0241 | 397.02249 | Unidentified |
| 5,681 | 567,2084 | 343.11887, 389.16129, 507.18716, 521.20264, 522.20709, 553.19464, 567.20801 | Unidentified |
| 5,684 | 521,2033 | 389.16147, 507.18698, 521.20227 | Unidentified |
| 5,698 | 507,188 | 507.18732 | Unidentified |
| 5,698 | 553,1939 | 507.18732, 553.19446 | Unidentified |
| 5,705 | 397,0241 | 397.02545 | Unidentified |
| 5,733 | 331,0125 | 205.98537, 251.05652, 331.01389 | Unidentified |
| 5,774 | 397,0244 | 397.02255 | Unidentified |
| 5,813 | 316,9972 | 206.99339, 316.99704 | Identified |
| 5,829 | 251,0558 | 205.98557, 221.00877, 251.05551 | Unidentified |
| 5,829 | 331,0132 | 205.98557, 221.00877, 251.05551, 331.01239 | Unidentified |
| 5,858 | 316,9983 | ND | Unidentified |
| 5,905 | 237,0407 | 190.99886, 221.04684 | Unidentified |
| 5,909 | 316,9975 | 206.99327 | Unidentified |
| 5,964 | 261,135 | 163.00386, 190.99893, 221.04599, 222.04866 | Unidentified |
| 5,968 | 190,9985 | 163.00377, 164.00728, 190.99869 | Unidentified |
| 5,968 | 620,0396 | 163.00377, 164.00728, 190.99869, 192.00214, 193.00455, 206.02243, 221.04587, 222.04961, 301.00238, 302.0054, 303.00204, 318.94379, 320.94138, 385.98157, 398.90192, 400.8992, 465.07986, 545.03656, 546.04102, 609.02051 | Unidentified |
| 5,974 | 206,0222 | 163.00377, 164.00728, 190.99869, 192.00214, 193.00455, 206.02243 | Unidentified |
| 5,975 | 640,9681 | 163.00397, 164.00742, 190.99879, 192.00186, 193.00398, 206.02179, 221.04568, 222.04852, 301.00241, 302.00565, 303.00113, 318.94431, 320.9425, 385.98029, 398.90274, 465.07919, 545.03674, 546.03882, 609.02032, 620.03723, 624.9939, 625.9978, 626.99396, 627.99658, 640.9682 | Unidentified |
| 5,976 | 624,9938 | 163.00377, 164.00728, 190.99869, 192.00214, 193.00455, 206.02243, 221.04587, 222.04961, 301.00238, 302.0054, 303.00204, 318.94379, 320.94138, 385.98157, 398.90192, 400.8992, 465.07986, 545.03656, 546.04102, 609.02051, 624.99396 | Unidentified |
| 5,978 | 221,0456 | 163.00377, 164.00728, 190.99869, 192.00214, 193.00455, 206.02243, 221.04587 | Unidentified |
| 6,001 | 301,0024 | 163.00377, 164.00728, 190.99869, 192.00214, 193.00455, 206.02243, 221.04587, 222.04961, 301.00238 | Identified |
| 5,991 | 237,0411 | 163.00378, 164.00677, 178.99811, 192.00206, 206.02187, 206.99335, 207.99715, 221.04565, 222.01733, 222.04909 | Unidentified |
| 5,994 | 316,9979 | 163.00378, 164.00677, 178.99811, 190.99863, 192.00206, 193.00365, 206.99335, 207.99715, 222.01733, 237.03978, 316.9978 | Unidentified |
| 6,005 | 554,262 | 163.00481, 190.9984, 192.0013, 206.99321, 207.99623, 222.01744, 237.04044, 238.04401, 316.99722, 318.99625, 338.97946, 381.96451, 400.95319, 416.92126 | Unidentified |
| 6,022 | 624,9952 | 163.00417, 164.00682, 192.0024, 206.02203, 221.04584, 222.049, 302.00613, 303.00183, 385.98123, 465.08054, 546.04456, 609.02344, 624.9953 | Unidentified |
| 6,03 | 190,9992 | 145.93132, 174.95619, 190.99904 | Unidentified |
| 6,04 | 163,004 | 145.93124, 163.00403 | Unidentified |
| 6,048 | 206,9936 | 145.93088, 174.95618, 190.99867, 206.02106, 206.99347 | Unidentified |
| 6,053 | 221,0457 | 145.93227, 174.95686, 190.99886, 206.02228, 221.04585 | Identified |
| 6,069 | 624,9946 | 145.93047, 163.00433, 190.99855, 206.02223, 221.04556, 222.04863, 223.05098, 235.92744, 237.04074, 301.00235, 302.00555, 303.00101, 304.00394, 318.00171, 366.9765, 368.99146, 385.97922, 411.97427, 413.01562, 425.05597, 430.95975, 569.22565, 624.99384 | Unidentified |
| 6,079 | 163,0039 | 145.93137, 163.004 | Unidentified |
| 6,079 | 190,9989 | 145.93047, 163.00433, 190.99855 | Unidentified |
| 6,088 | 316,9969 | 145.93195, 146.93845, 174.95602, 190.99889, 206.9944, 221.04611, 222.01743, 222.04877, 235.9263, 237.04092, 238.04443, 301.00275, 302.00641, 303.00195, 316.99771 | Unidentified |
| 6,11 | 206,9937 | 145.93138, 174.9557, 190.9995, 206.99347 | Unidentified |
| 6,138 | 316,9977 | 206.99361, 222.01692, 237.04062, 238.04414, 301.00247, 316.99734 | Unidentified |
| 6,156 | 237,0407 | 145.93155, 190.99875, 206.99333, 221.04565, 222.01709, 235.9263, 237.04042 | Identified |
| 6,158 | 206,9942 | 145.93195, 190.9994, 206.99422 | Unidentified |
| 6,847 | 190,999 | 163.00395, 190.99876 | Unidentified |
| 6,847 | 221,046 | 163.00395, 190.99876, 192.0024, 206.02199, 221.04565 | Identified |
| 6,854 | 719,0714 | 163.00412, 190.99881, 192.00206, 193.00385, 205.01472, 206.02229, 207.02609, 221.04587, 221.20834, 222.04927, 223.05125, 284.98654, 289.03296, 306.02341, 318.94473, 320.94061, 320.97128, 321.97485, 322.97733, 333.05933, 351.0051, 360.96677, 366.97937, 368.97687, 383.98013, 385.9769, 409.99155, 411.97458, 412.97653, 449.10947, 465.08142, 466.0867, 481.05334, 505.02234, 526.02185, 527.02631, 528.02527, 543.02338, 544.02826, 545.02533, 546.026, 547.02094, 560.02313, 562.02472, 567.96466, 569.95776, 577.99231, 586.03802, 588.02179, 589.02722, 590.02502, 613.9505, 638.00696, 709.11462, 710.12018, 719.07245 | Unidentified |
| 6,854 | 940,1166 | 163.00412, 190.99881, 192.00206, 193.00385, 205.01472, 206.02229, 207.02609, 221.04587, 221.20834, 222.04927, 223.05125, 284.98654, 289.03296, 306.02341, 318.94473, 320.94061, 320.97128, 321.97485, 322.97733, 333.05933, 351.0051, 360.96677, 366.97937, 368.97687, 383.98013, 385.9769, 409.99155, 411.97458, 412.97653, 449.10947, 465.08142, 466.0867, 481.05334, 505.02234, 526.02185, 527.02631, 528.02527, 543.02338, 544.02826, 545.02533, 546.026, 547.02094, 560.02313, 562.02472, 567.96466, 569.95776, 577.99231, 586.03802, 588.02179, 589.02722, 590.02502, 613.9505, 638.00696, 709.11462, 710.12018, 719.07245, 721.07159, 738.07007, 739.07526, 754.04034, 755.0412, 756.04083, 757.03833, 764.06976, 765.07135, 814.05133, 832.05414, 938.12036, 939.13263, 940.11652 | Unidentified |
| 6,855 | 320,9701 | 163.00412, 190.99881, 192.00206, 193.00385, 205.01472, 206.02229, 207.02609, 221.04587, 221.20834, 222.04927, 223.05125, 284.98654, 289.03296, 306.02341, 318.94473, 320.94061, 320.97128 | Unidentified |
| 6,855 | 465,0808 | 163.00412, 190.99881, 192.00206, 193.00385, 205.01472, 206.02229, 207.02609, 221.04587, 221.20834, 222.04927, 223.05125, 284.98654, 289.03296, 306.02341, 318.94473, 320.94061, 320.97128, 321.97485, 322.97733, 333.05933, 351.0051, 360.96677, 366.97937, 368.97687, 383.98013, 385.9769, 409.99155, 411.97458, 412.97653, 449.10947, 465.08142 | Unidentified |
| 6,858 | 163,004 | 163.00412 | Unidentified |
| 7,083 | 583,1679 | 429.10422, 583.16589 | Unidentified |
| 7,166 | 585,2344 | 537.216, 585.23206 | Identified |
| 7,291 | 695,4006 | 487.34286, 488.34698, 695.40112 | Unidentified |
| 7,346 | 585,2347 | 585.23315 | Unidentified |
| 7,839 | 215,1288 | ND | Unidentified |
| 7,985 | 599,2504 | 329.1037, 341.10416, 344.12607, 356.12683, 359.1488, 371.15155, 415.1821, 599.24847 | Unidentified |
| 8,005 | 554,262 | 145.9314, 174.95607, 235.92659, 325.18396, 415.18106, 416.18277 | Unidentified |
| 8,108 | 415,1798 | 145.93098, 174.9566, 235.92709, 413.16379, 415.17957 | Unidentified |
| 8,453 | 401,201 | 311.16998, 325.18283, 329.23441, 401.20117 | Unidentified |
| 8,509 | 415,1802 | 235.92667, 325.18497, 329.23257, 341.11234, 402.20557, 415.18015 | Unidentified |
| 8,564 | 415,1794 | 229.14516, 235.92595, 415.17987 | Unidentified |
| 8,624 | 415,1798 | 235.92589, 311.16885, 401.20261, 415.17978 | Unidentified |
| 9,048 | 315,1816 | ND | Unidentified |
| 9,249 | 387,287 | ND | Unidentified |
| 9,342 | 399,1858 | 399.18469 | Unidentified |
| 9,581 | 236,1056 | 221.15463 | Unidentified |
| 9,587 | 293,1758 | 221.15347, 279.23361 | Unidentified |
| 9,867 | 399,1857 | 279.23462, 353.18008, 399.18533 | Unidentified |
| 9,867 | 601,3239 | 279.23257, 399.18521, 400.19, 401.18665, 421.16763, 601.32288 | Unidentified |
| 9,879 | 467,1724 | 279.23462, 353.18008, 399.18533, 400.18918, 401.18512, 421.16891 | Unidentified |
| 9,922 | 343,2129 | 325.18442 | Unidentified |
| 9,922 | 399,1852 | 399.18478 | Unidentified |
| 9,974 | 399,1856 | 183.01279, 281.24899, 325.18527, 399.18527 | Unidentified |
| 10,006 | 554,262 | 287.22299, 311.1687, 325.18411, 339.20016, 343.21237, 377.27551, 399.18597, 400.18805 | Unidentified |
| 10,37 | 399,1848 | 340.20251, 399.18567 | Unidentified |
| 10,425 | 399,1849 | 325.18539, 326.18707, 377.27365, 399.18521 | Unidentified |
| 10,521 | 399,1858 | 265.14783, 293.17993, 311.168, 315.25412, 325.185, 326.18719, 327.01624, 328.02002, 356.01712, 377.27316, 383.24213, 389.19861, 399.18521 | Unidentified |
| 10,575 | 399,1851 | 157.01193, 201.03848, 315.25305, 325.18396, 339.19839, 399.1853 | Unidentified |
| 11,046 | 621,437 | 183.01329, 201.03833, 255.23257, 279.23364, 280.23672, 281.24817, 325.18344, 339.20151, 391.23724, 621.43707 | Unidentified |
| 11,091 | 221,1545 | 183.01297, 201.03903 | Unidentified |
| 11,113 | 621,4372 | 201.03906, 279.23257, 339.19989, 621.4353 | Unidentified |
| 11,166 | 621,4365 | 201.03725, 279.23328, 621.43707 | Unidentified |
| 11,345 | 297,2432 | 183.01221, 201.0376, 255.23184, 279.23209, 281.2478 | Unidentified |
| 11,69 | 458,421 | 183.0123, 201.03792, 255.23398, 279.23257, 280.23727, 281.24902, 325.18298, 339.20187, 391.23383, 431.1944 | Unidentified |
| 11,772 | 458,4219 | 201.03873, 255.23366, 279.23318, 281.24768, 339.19897, 391.23041, 431.19354 | Unidentified |
| 11,781 | 250,1451 | 183.01175, 201.03911 | Unidentified |

ND- Not detected


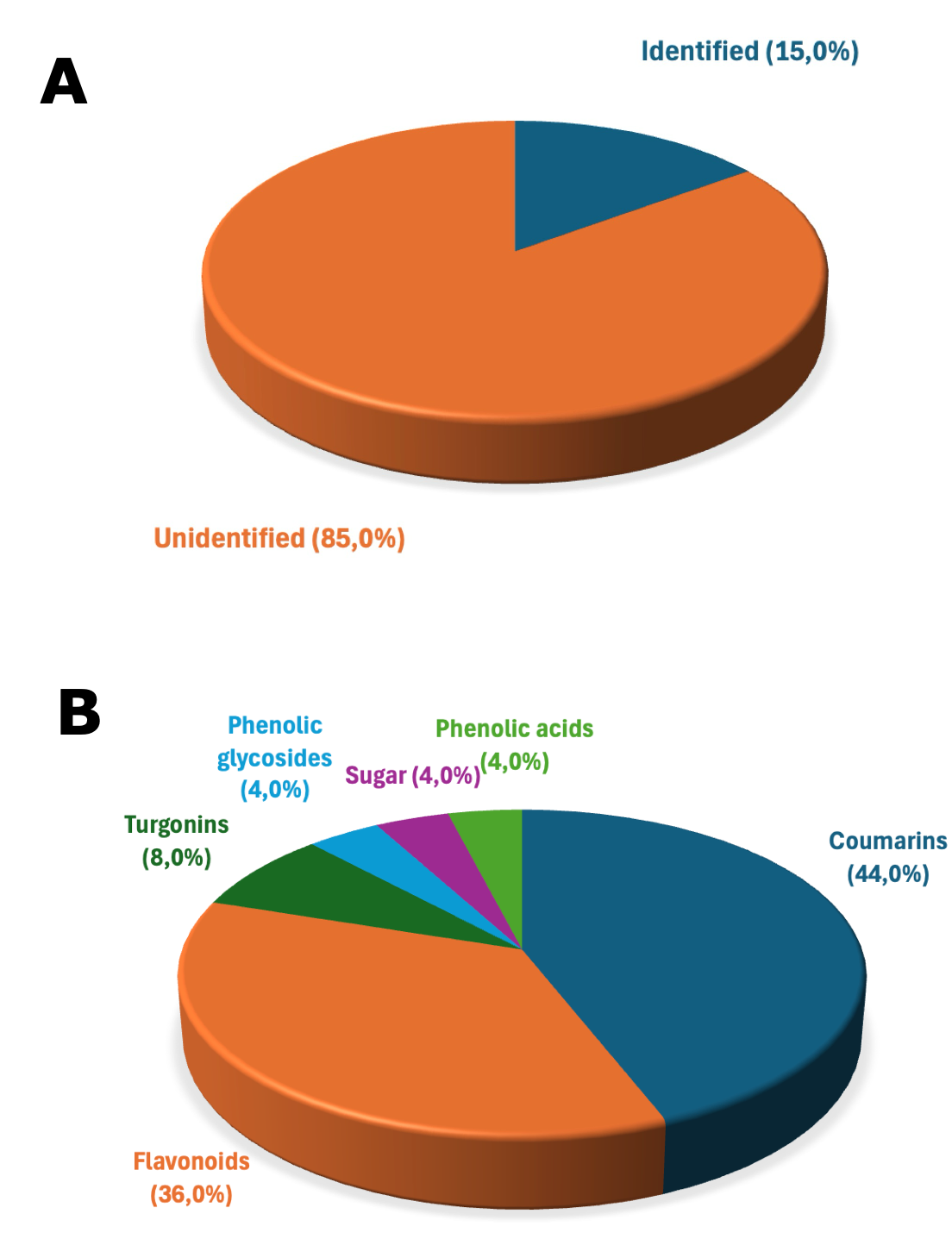


**Fig. S4** Overview of identified metabolites in obtained features (a) and groups of identified metabolites (b).

**Table S2:** OPLS-DA descriptive statistics for 6 vs 12 months harvest age.

| **Var_ID** | **Probability** | **Fold_change** | **Significant_raw** | **FDR_BH** | **Significant_FDR** |
| --- | --- | --- | --- | --- | --- |
| 1,224/387,11526 | 0,000256 | 0,234283 | TRUE | 0,002356 | TRUE |
| 1,225/440,02698 | 0,000234 | 0,259918 | TRUE | 0,002356 | TRUE |
| 1,226/341,10965 | 0,000242 | 0,24312 | TRUE | 0,002356 | TRUE |
| 1,226/439,08325 | 0,000744 | 0,169704 | TRUE | 0,005067 | TRUE |
| 1,226/736,13794 | 0,00466 | 0,046489 | TRUE | 0,022214 | TRUE |
| 1,227/683,22571 | 0,007464 | 0,038309 | TRUE | 0,028847 | TRUE |
| 1,23/377,08719 | 0,000264 | 0,153008 | TRUE | 0,002356 | TRUE |
| 1,236/711,22186 | 0,926328 | 1,0201 | FALSE | 0,952985 | FALSE |
| 1,239/549,16864 | 9,81E-07 | 0,304886 | TRUE | 3,51E-05 | TRUE |
| 2,004/554,26202 | 0,000514 | 0,937092 | TRUE | 0,003672 | TRUE |
| 2,887/216,03371 | 0,212087 | 11,5424 | FALSE | 0,328596 | FALSE |
| 2,911/328,04596 | 0,230844 | 0 | FALSE | 0,347481 | FALSE |
| 3,024/169,01474 | 4,01E-07 | 7,31748 | TRUE | 1,91E-05 | TRUE |
| 3,028/609,12549 | 0,008191 | 2,19529 | TRUE | 0,030825 | TRUE |
| 3,094/331,10373 | 0,00221 | 2,19467 | TRUE | 0,012639 | TRUE |
| 3,16/358,05682 | 0,182862 | 0,423218 | FALSE | 0,293812 | FALSE |
| 3,189/913,18549 | 0,085683 | 1,54849 | FALSE | 0,1616 | FALSE |
| 3,281/411,02463 | 0,556605 | 1,17084 | FALSE | 0,668675 | FALSE |
| 3,29/609,12585 | 0,025497 | 1,59636 | TRUE | 0,07596 | FALSE |
| 3,353/272,97116 | 0,945659 | 1,02582 | FALSE | 0,953499 | FALSE |
| 3,383/913,18378 | 0,376716 | 1,20932 | FALSE | 0,4988 | FALSE |
| 3,429/272,97162 | 0,323676 | 1,44838 | FALSE | 0,449375 | FALSE |
| 3,476/305,06693 | 0,006001 | 2,30984 | TRUE | 0,025239 | TRUE |
| 3,477/913,18591 | 0,630366 | 1,12678 | FALSE | 0,738872 | FALSE |
| 3,512/760,14563 | 0,765862 | 1,05454 | FALSE | 0,840782 | FALSE |
| 3,527/608,11743 | 0,561126 | 1,11569 | FALSE | 0,668675 | FALSE |
| 3,54/913,18176 | 0,441823 | 1,1781 | FALSE | 0,554819 | FALSE |
| 3,559/609,12616 | 0,337833 | 1,29496 | FALSE | 0,455756 | FALSE |
| 3,618/593,13202 | 0,831807 | 0,943124 | FALSE | 0,894349 | FALSE |
| 3,947/305,06738 | 0,068845 | 1,61064 | FALSE | 0,147607 | FALSE |
| 3,948/404,99182 | 0,084785 | 1,63456 | FALSE | 0,1616 | FALSE |
| 3,948/593,13147 | 0,029131 | 2,75919 | TRUE | 0,080305 | FALSE |
| 3,953/611,14172 | 0,067229 | 5,01218 | FALSE | 0,147607 | FALSE |
| 3,959/193,01456 | 0,01169 | 2,31102 | TRUE | 0,0398 | TRUE |
| 3,961/373,0549 | 0,07521 | 1,69152 | FALSE | 0,152976 | FALSE |
| 3,961/449,99133 | 0,045837 | 1,8341 | TRUE | 0,111097 | FALSE |
| 3,962/303,05124 | 0,038737 | 1,9611 | TRUE | 0,098917 | FALSE |
| 3,963/272,97141 | 0,013147 | 2,13549 | TRUE | 0,04372 | TRUE |
| 3,964/365,01935 | 0,36481 | 1,92929 | FALSE | 0,48755 | FALSE |
| 4,006/554,26202 | 0,166445 | 0,977383 | FALSE | 0,280019 | FALSE |
| 4,91/270,99237 | 0,67415 | 1,13474 | FALSE | 0,771228 | FALSE |
| 4,963/270,99228 | 0,056262 | 2,9499 | FALSE | 0,131893 | FALSE |
| 5,018/270,992 | 0,153538 | NFC | FALSE | 0,267623 | FALSE |
| 5,031/383,00876 | 0,044377 | 2,71282 | TRUE | 0,109412 | FALSE |
| 5,072/207,03032 | 0,107087 | 3,96573 | FALSE | 0,196326 | FALSE |
| 5,113/575,1629 | 0,181021 | 2,14538 | FALSE | 0,293812 | FALSE |
| 5,128/383,00824 | 0,43686 | 1,3127 | FALSE | 0,554819 | FALSE |
| 5,184/383,00821 | 0,029202 | 3,46214 | TRUE | 0,080305 | FALSE |
| 5,195/773,25208 | 0,529938 | 1,00906 | FALSE | 0,647702 | FALSE |
| 5,204/301,00241 | 0,256281 | 2,89827 | FALSE | 0,381752 | FALSE |
| 5,226/429,1051 | 0,203823 | 3,44526 | FALSE | 0,323852 | FALSE |
| 5,254/581,22388 | 0,660355 | 0,863811 | FALSE | 0,76773 | FALSE |
| 5,295/537,19897 | 0,032641 | 3,05683 | TRUE | 0,086438 | FALSE |
| 5,295/583,20551 | 0,040825 | 2,73564 | TRUE | 0,10242 | FALSE |
| 5,316/206,0824 | 0,889975 | 0,836299 | FALSE | 0,92222 | FALSE |
| 5,386/173,08189 | 0,001401 | 1,133 | TRUE | 0,008708 | TRUE |
| 5,393/551,21356 | 0,756972 | 0,879078 | FALSE | 0,840782 | FALSE |
| 5,394/597,22021 | 0,670191 | 0,818282 | FALSE | 0,771228 | FALSE |
| 5,401/367,01315 | 0,692934 | 1,18932 | FALSE | 0,786425 | FALSE |
| 5,465/367,01379 | 0,085194 | 2,43592 | FALSE | 0,1616 | FALSE |
| 5,482/242,99739 | 0,335057 | 0,625432 | FALSE | 0,455756 | FALSE |
| 5,556/242,99709 | 0,760133 | 1,32159 | FALSE | 0,840782 | FALSE |
| 5,563/367,01312 | 0,552755 | 1,30459 | FALSE | 0,668675 | FALSE |
| 5,569/551,21313 | 0,848917 | 0,943147 | FALSE | 0,899223 | FALSE |
| 5,58/597,21899 | 0,953499 | 1,02013 | FALSE | 0,953499 | FALSE |
| 5,617/787,26733 | 0,024263 | 1,02882 | TRUE | 0,073928 | FALSE |
| 5,633/397,02414 | 0,475818 | 1,61685 | FALSE | 0,586569 | FALSE |
| 5,681/567,20837 | 0,829309 | 0,91007 | FALSE | 0,894349 | FALSE |
| 5,684/521,20331 | 0,58964 | 0,883745 | FALSE | 0,696847 | FALSE |
| 5,698/507,18796 | 0,770227 | 1,13798 | FALSE | 0,840782 | FALSE |
| 5,698/553,19385 | 0,838597 | 1,0983 | FALSE | 0,894921 | FALSE |
| 5,705/397,02411 | 0,284486 | 2,18943 | FALSE | 0,415117 | FALSE |
| 5,733/331,01248 | 0,405721 | 0,733721 | FALSE | 0,527437 | FALSE |
| 5,774/397,02444 | 0,443658 | 1,68485 | FALSE | 0,554819 | FALSE |
| 5,813/316,99716 | 0,068171 | 0,606398 | FALSE | 0,147607 | FALSE |
| 5,858/316,99826 | 0,942171 | 0,977788 | FALSE | 0,953499 | FALSE |
| 5,905/237,04066 | 0,000404 | 2,7794 | TRUE | 0,003195 | TRUE |
| 5,909/316,99753 | 0,156482 | 8,24098 | FALSE | 0,267623 | FALSE |
| 5,964/261,13504 | 1,62E-08 | 1,41892 | TRUE | 1,16E-06 | TRUE |
| 5,968/190,9985 | 0,028917 | 0,629197 | TRUE | 0,080305 | FALSE |
| 5,968/620,03961 | 0,211539 | 0,330153 | FALSE | 0,328596 | FALSE |
| 5,974/206,02219 | 0,075953 | 0,698675 | FALSE | 0,152976 | FALSE |
| 5,975/640,96814 | 0,157205 | 0,588347 | FALSE | 0,267623 | FALSE |
| 5,976/624,99384 | 0,181154 | 0,531955 | FALSE | 0,293812 | FALSE |
| 5,978/221,04564 | 0,069568 | 0,705831 | FALSE | 0,147607 | FALSE |
| 6,001/301,00244 | 0,07167 | 0,713327 | FALSE | 0,148533 | FALSE |
| 5,991/237,04105 | 0,006925 | 0,238705 | TRUE | 0,027506 | TRUE |
| 5,994/316,99786 | 0,003208 | 0,166551 | TRUE | 0,016386 | TRUE |
| 6,005/554,26202 | 0,024298 | 0,958646 | TRUE | 0,073928 | FALSE |
| 6,022/624,99518 | 0,87924 | 1,0546 | FALSE | 0,921153 | FALSE |
| 6,03/190,99915 | 0,051763 | 1,66977 | FALSE | 0,123368 | FALSE |
| 6,04/163,00395 | 0,032464 | 1,8958 | TRUE | 0,086438 | FALSE |
| 6,048/206,99362 | 0,882503 | 0,965577 | FALSE | 0,921153 | FALSE |
| 6,053/221,04573 | 0,009168 | 1,81131 | TRUE | 0,03278 | TRUE |
| 6,069/624,99457 | 0,128009 | 53,4221 | FALSE | 0,228816 | FALSE |
| 6,079/163,00394 | 0,009169 | 8,01166 | TRUE | 0,03278 | TRUE |
| 6,079/190,99886 | 0,003496 | 4,89922 | TRUE | 0,017238 | TRUE |
| 6,088/316,99689 | 0,337315 | 1,2884 | FALSE | 0,455756 | FALSE |
| 6,11/206,9937 | 0,000984 | 5,68543 | TRUE | 0,006397 | TRUE |
| 6,138/316,99765 | 0,082514 | 27,7976 | FALSE | 0,1616 | FALSE |
| 6,158/206,99416 | 0,069729 | 13,8264 | FALSE | 0,147607 | FALSE |
| 6,847/190,99902 | 0,002601 | 2,54756 | TRUE | 0,013778 | TRUE |
| 6,847/221,04597 | 0,000425 | 2,25609 | TRUE | 0,003195 | TRUE |
| 6,854/719,07135 | 0,174308 | 4,37297 | FALSE | 0,289838 | FALSE |
| 6,854/940,11664 | 0,085886 | 7,02041 | FALSE | 0,1616 | FALSE |
| 6,855/320,97009 | 0,022551 | NFC | TRUE | 0,071662 | FALSE |
| 6,855/465,08078 | 0,005698 | 7,90521 | TRUE | 0,024692 | TRUE |
| 6,858/163,004 | 0,125434 | 4,42675 | FALSE | 0,227051 | FALSE |
| 7,166/585,23438 | 0,000342 | 0,351204 | TRUE | 0,002881 | TRUE |
| 7,291/695,40063 | 0,027283 | 4,35734 | TRUE | 0,079621 | FALSE |
| 7,346/585,23474 | 0,000135 | 0,351705 | TRUE | 0,001607 | TRUE |
| 7,839/215,1288 | 0,006379 | 1,0683 | TRUE | 0,026064 | TRUE |
| 7,985/599,25043 | 0,446183 | 0,650307 | FALSE | 0,554819 | FALSE |
| 8,005/554,26202 | 0,066191 | 0,963742 | FALSE | 0,147607 | FALSE |
| 8,108/415,17975 | 0,070191 | 2,05566 | FALSE | 0,147607 | FALSE |
| 8,453/401,20099 | 0,401538 | 1,35807 | FALSE | 0,526788 | FALSE |
| 8,509/415,18015 | 0,414988 | 0,807504 | FALSE | 0,534624 | FALSE |
| 8,564/415,17935 | 0,002316 | 2,33566 | TRUE | 0,012739 | TRUE |
| 8,624/415,17978 | 0,005053 | 3,44168 | TRUE | 0,023308 | TRUE |
| 9,048/315,18155 | 5,11E-06 | 0,864482 | TRUE | 0,000146 | TRUE |
| 9,249/387,28702 | 0,00012 | 1,15229 | TRUE | 0,001566 | TRUE |
| 9,342/399,18576 | 0,3099 | 1,32382 | FALSE | 0,434468 | FALSE |
| 9,581/236,10558 | 0,217648 | 1,10054 | FALSE | 0,331103 | FALSE |
| 9,587/293,17584 | 0,130891 | 1,11935 | FALSE | 0,231079 | FALSE |
| 9,867/399,18567 | 0,27518 | 0,777608 | FALSE | 0,405678 | FALSE |
| 9,867/601,32385 | 1,9E-05 | 0,84421 | TRUE | 0,000389 | TRUE |
| 9,879/467,17239 | 0,30462 | 0,792772 | FALSE | 0,433427 | FALSE |
| 9,922/343,21289 | 1,08E-05 | 0,890126 | TRUE | 0,000258 | TRUE |
| 9,922/399,18521 | 0,09841 | 1,60808 | FALSE | 0,182762 | FALSE |
| 9,974/399,18561 | 0,00544 | 4,33787 | TRUE | 0,024308 | TRUE |
| 10,006/554,26202 | 0,00178 | 0,927564 | TRUE | 0,010605 | TRUE |
| 10,37/399,18481 | 0,949248 | 1,02417 | FALSE | 0,953499 | FALSE |
| 10,425/399,18491 | 0,294533 | 1,64284 | FALSE | 0,425437 | FALSE |
| 10,521/399,18579 | 0,036002 | 25,3312 | TRUE | 0,093606 | FALSE |
| 10,575/399,18509 | 0,213702 | NFC | FALSE | 0,328596 | FALSE |
| 11,046/621,43701 | 0,010814 | 0,61024 | TRUE | 0,037716 | TRUE |
| 11,091/221,15454 | 0,306127 | 1,01747 | FALSE | 0,433427 | FALSE |
| 11,113/621,43719 | 0,000104 | 0,413722 | TRUE | 0,001493 | TRUE |
| 11,166/621,43652 | 6,42E-05 | 0,327498 | TRUE | 0,00102 | TRUE |
| 11,345/297,24319 | 0,021519 | 1,06002 | TRUE | 0,069936 | FALSE |
| 11,69/458,42099 | 3,42E-05 | 2,3695 | TRUE | 0,000612 | TRUE |
| 11,772/458,42194 | 8,38E-12 | 0,451296 | TRUE | 1,2E-09 | TRUE |
| 11,781/250,14514 | 0,728023 | 0,993828 | FALSE | 0,819742 | FALSE |

Var_ID , Variable identification; NFC= No fold change; FDR_BH,False Discovery Rate -Benjamini-Hochberg

**Table S3:** OPLS-DA descriptive statistics for 6 *versus* 18 months harvest age.

| **Var_ID** | **Probability** | **Fold_change** | **Significant_raw** | **FDR_BH** | **Significant_FDR** |
| --- | --- | --- | --- | --- | --- |
| 1,224/387,11526 | 0,03349 | 0,536076 | TRUE | 0,054562 | FALSE |
| 1,225/440,02698 | 0,051462 | 0,592801 | FALSE | 0,077729 | FALSE |
| 1,226/341,10965 | 0,041851 | 0,560052 | TRUE | 0,065252 | FALSE |
| 1,226/439,08325 | 0,046926 | 0,473804 | TRUE | 0,072385 | FALSE |
| 1,226/736,13794 | 0,085863 | 0,377561 | FALSE | 0,117454 | FALSE |
| 1,227/683,22571 | 0,111396 | 0,393253 | FALSE | 0,144218 | FALSE |
| 1,23/377,08719 | 0,094048 | 0,560899 | FALSE | 0,126268 | FALSE |
| 1,236/711,22186 | 0,137413 | 1,50374 | FALSE | 0,168855 | FALSE |
| 1,239/549,16864 | 0,000851 | 0,536795 | TRUE | 0,002868 | TRUE |
| 2,004/554,26202 | 4,32E-05 | 0,920343 | TRUE | 0,000255 | TRUE |
| 2,887/216,03371 | 0,27951 | 8,48011 | FALSE | 0,321658 | FALSE |
| 2,911/328,04596 | 0,532035 | 0,470793 | FALSE | 0,563103 | FALSE |
| 3,024/169,01474 | 0,011021 | 4,23578 | TRUE | 0,022195 | TRUE |
| 3,028/609,12549 | 0,003023 | 2,20468 | TRUE | 0,007306 | TRUE |
| 3,094/331,10373 | 0,000281 | 3,81074 | TRUE | 0,001133 | TRUE |
| 3,16/358,05682 | 0,725914 | 0,839108 | FALSE | 0,757248 | FALSE |
| 3,189/913,18549 | 0,032252 | 1,56365 | TRUE | 0,053753 | FALSE |
| 3,281/411,02463 | 0,799896 | 1,06024 | FALSE | 0,81587 | FALSE |
| 3,29/609,12585 | 0,002537 | 1,93885 | TRUE | 0,006236 | TRUE |
| 3,353/272,97116 | 0,397512 | 0,698663 | FALSE | 0,436661 | FALSE |
| 3,383/913,18378 | 0,031458 | 1,56754 | TRUE | 0,053039 | FALSE |
| 3,429/272,97162 | 0,017286 | 2,65393 | TRUE | 0,032551 | TRUE |
| 3,476/305,06693 | 0,003756 | 1,93813 | TRUE | 0,008927 | TRUE |
| 3,477/913,18591 | 0,078962 | 1,74158 | FALSE | 0,111617 | FALSE |
| 3,512/760,14563 | 0,091395 | 1,37964 | FALSE | 0,123853 | FALSE |
| 3,527/608,11743 | 0,02033 | 1,57692 | TRUE | 0,037314 | TRUE |
| 3,54/913,18176 | 0,060699 | 1,57347 | FALSE | 0,088516 | FALSE |
| 3,559/609,12616 | 0,034565 | 1,73352 | TRUE | 0,055688 | FALSE |
| 3,618/593,13202 | 0,181001 | 1,46469 | FALSE | 0,215124 | FALSE |
| 3,947/305,06738 | 0,000959 | 2,05038 | TRUE | 0,003161 | TRUE |
| 3,948/404,99182 | 0,018818 | 1,60805 | TRUE | 0,034983 | TRUE |
| 3,948/593,13147 | 0,001552 | 4,67381 | TRUE | 0,004329 | TRUE |
| 3,953/611,14172 | 0,001605 | 7,04467 | TRUE | 0,004392 | TRUE |
| 3,959/193,01456 | 0,395611 | 1,34461 | FALSE | 0,436661 | FALSE |
| 3,961/373,0549 | 0,001298 | 2,14972 | TRUE | 0,003842 | TRUE |
| 3,961/449,99133 | 0,001344 | 2,28466 | TRUE | 0,003898 | TRUE |
| 3,962/303,05124 | 0,000221 | 2,85877 | TRUE | 0,000917 | TRUE |
| 3,963/272,97141 | 0,450673 | 1,2635 | FALSE | 0,491335 | FALSE |
| 3,964/365,01935 | 0,100238 | 3,38146 | FALSE | 0,130942 | FALSE |
| 4,006/554,26202 | 2,22E-05 | 0,90322 | TRUE | 0,000161 | TRUE |
| 4,91/270,99237 | 0,035656 | 0,477762 | TRUE | 0,056815 | FALSE |
| 4,963/270,99228 | 0,000395 | 3,44598 | TRUE | 0,001507 | TRUE |
| 5,018/270,992 | 0,001269 | NFC | TRUE | 0,003833 | TRUE |
| 5,031/383,00876 | 0,014812 | 23,1438 | TRUE | 0,028334 | TRUE |
| 5,072/207,03032 | 0,315441 | 1,64122 | FALSE | 0,360149 | FALSE |
| 5,113/575,1629 | 0,32845 | 1,70249 | FALSE | 0,372072 | FALSE |
| 5,128/383,00824 | 0,784779 | 0,894457 | FALSE | 0,812807 | FALSE |
| 5,184/383,00821 | 0,001826 | 9,72872 | TRUE | 0,004815 | TRUE |
| 5,195/773,25208 | 0,000343 | 1,07096 | TRUE | 0,001344 | TRUE |
| 5,204/301,00241 | 0,980508 | 1,0165 | FALSE | 0,980508 | FALSE |
| 5,226/429,1051 | 0,255533 | 1,92715 | FALSE | 0,296418 | FALSE |
| 5,254/581,22388 | 0,098659 | 2,03385 | FALSE | 0,130051 | FALSE |
| 5,277/301,00323 | 0,024884 | NFC | TRUE | 0,044003 | TRUE |
| 5,295/537,19897 | 0,029121 | 5,20377 | TRUE | 0,049676 | TRUE |
| 5,295/583,20551 | 0,025333 | 4,65715 | TRUE | 0,044256 | TRUE |
| 5,316/206,0824 | 0,367898 | 3,19065 | FALSE | 0,410348 | FALSE |
| 5,386/173,08189 | 7,74E-08 | 1,33879 | TRUE | 1,12E-06 | TRUE |
| 5,393/551,21356 | 0,122328 | 2,14345 | FALSE | 0,15339 | FALSE |
| 5,394/597,22021 | 0,141581 | 2,15942 | FALSE | 0,172515 | FALSE |
| 5,401/367,01315 | 0,061046 | 0,357002 | FALSE | 0,088516 | FALSE |
| 5,465/367,01379 | 0,008348 | 5,06637 | TRUE | 0,017542 | TRUE |
| 5,482/242,99739 | 0,079286 | 0,332426 | FALSE | 0,111617 | FALSE |
| 5,556/242,99709 | 0,020924 | 99,0793 | TRUE | 0,037925 | TRUE |
| 5,563/367,01312 | 0,149628 | 0,50873 | FALSE | 0,179306 | FALSE |
| 5,569/551,21313 | 0,135827 | 1,94408 | FALSE | 0,168333 | FALSE |
| 5,58/597,21899 | 0,117419 | 1,9786 | FALSE | 0,149349 | FALSE |
| 5,617/787,26733 | 1,67E-06 | 1,09012 | TRUE | 1,86E-05 | TRUE |
| 5,633/397,02414 | 0,800111 | 1,27101 | FALSE | 0,81587 | FALSE |
| 5,681/567,20837 | 0,0537 | 2,97942 | FALSE | 0,080274 | FALSE |
| 5,684/521,20331 | 0,095922 | 2,43931 | FALSE | 0,127603 | FALSE |
| 5,698/507,18796 | 0,084471 | 5,81736 | FALSE | 0,11665 | FALSE |
| 5,698/553,19385 | 0,081088 | 5,8372 | FALSE | 0,113056 | FALSE |
| 5,705/397,02411 | 0,047781 | 14,2066 | TRUE | 0,072929 | FALSE |
| 5,733/331,01248 | 0,037295 | 0,321876 | TRUE | 0,058781 | FALSE |
| 5,774/397,02444 | 0,507358 | 1,70267 | FALSE | 0,540933 | FALSE |
| 5,813/316,99716 | 9,36E-05 | 0,121857 | TRUE | 0,000452 | TRUE |
| 5,829/251,0558 | 0,033045 | NFC | TRUE | 0,054449 | FALSE |
| 5,829/331,01318 | 0,014851 | NFC | TRUE | 0,028334 | TRUE |
| 5,858/316,99826 | 0,576409 | 0,825218 | FALSE | 0,605647 | FALSE |
| 5,905/237,04066 | 0,01153 | 2,12124 | TRUE | 0,022902 | TRUE |
| 5,909/316,99753 | 0,000129 | 49,6492 | TRUE | 0,000602 | TRUE |
| 5,964/261,13504 | 1E-16 | 1,73552 | TRUE | 1,46E-14 | TRUE |
| 5,968/190,9985 | 1,8E-06 | 0,070298 | TRUE | 1,86E-05 | TRUE |
| 5,968/620,03961 | 0,071399 | 0,050512 | FALSE | 0,102503 | FALSE |
| 5,974/206,02219 | 1,63E-05 | 0,125081 | TRUE | 0,000129 | TRUE |
| 5,975/640,96814 | 0,001211 | 0,053653 | TRUE | 0,003737 | TRUE |
| 5,976/624,99384 | 0,006313 | 0,052126 | TRUE | 0,013754 | TRUE |
| 5,978/221,04564 | 1,48E-05 | 0,16533 | TRUE | 0,000126 | TRUE |
| 6,001/301,00244 | 3,11E-05 | 0,259768 | TRUE | 0,000215 | TRUE |
| 5,991/237,04105 | 0,00112 | 0,070184 | TRUE | 0,00353 | TRUE |
| 5,994/316,99786 | 0,000595 | 0,016524 | TRUE | 0,002104 | TRUE |
| 6,005/554,26202 | 3,97E-05 | 0,920733 | TRUE | 0,00025 | TRUE |
| 6,022/624,99518 | 0,355226 | 1,41194 | FALSE | 0,399285 | FALSE |
| 6,03/190,99915 | 0,000551 | 2,69706 | TRUE | 0,001998 | TRUE |
| 6,04/163,00395 | 3,66E-06 | 3,52336 | TRUE | 3,54E-05 | TRUE |
| 6,048/206,99362 | 0,00428 | 0,255369 | TRUE | 0,009965 | TRUE |
| 6,053/221,04573 | 3,25E-10 | 3,8627 | TRUE | 1,18E-08 | TRUE |
| 6,069/624,99457 | 1,06E-05 | 213,066 | TRUE | 9,62E-05 | TRUE |
| 6,079/163,00394 | 4,81E-09 | 21,4704 | TRUE | 9,97E-08 | TRUE |
| 6,079/190,99886 | 3,61E-09 | 15,7184 | TRUE | 8,73E-08 | TRUE |
| 6,088/316,99689 | 0,804617 | 0,920921 | FALSE | 0,81587 | FALSE |
| 6,11/206,9937 | 5,49E-07 | 10,6294 | TRUE | 7,24E-06 | TRUE |
| 6,138/316,99765 | 1,17E-06 | 241,964 | TRUE | 1,42E-05 | TRUE |
| 6,156/237,04074 | 0,009226 | NFC | TRUE | 0,019112 | TRUE |
| 6,158/206,99416 | 0,000472 | 343,141 | TRUE | 0,001753 | TRUE |
| 6,847/190,99902 | 0,14534 | 1,59308 | FALSE | 0,175619 | FALSE |
| 6,847/221,04597 | 0,013624 | 1,72778 | TRUE | 0,026695 | TRUE |
| 6,854/940,11664 | 0,828674 | 0,746356 | FALSE | 0,834429 | FALSE |
| 6,855/320,97009 | 0,000157 | NFC | TRUE | 0,000689 | TRUE |
| 6,855/465,08078 | 0,236243 | 3,0237 | FALSE | 0,276252 | FALSE |
| 7,166/585,23438 | 0,022771 | 0,582622 | TRUE | 0,040762 | TRUE |
| 7,291/695,40063 | 0,058386 | 9,22789 | FALSE | 0,086388 | FALSE |
| 7,346/585,23474 | 0,010814 | 0,558331 | TRUE | 0,022086 | TRUE |
| 7,839/215,1288 | 0,001971 | 1,18396 | TRUE | 0,005105 | TRUE |
| 7,985/599,25043 | 0,486768 | 1,35597 | FALSE | 0,522825 | FALSE |
| 8,005/554,26202 | 0,002054 | 0,927372 | TRUE | 0,005226 | TRUE |
| 8,108/415,17975 | 0,234564 | 0,503669 | FALSE | 0,276252 | FALSE |
| 8,453/401,20099 | 0,006845 | 0,230128 | TRUE | 0,014596 | TRUE |
| 8,509/415,18015 | 0,001548 | 0,210724 | TRUE | 0,004329 | TRUE |
| 8,564/415,17935 | 0,027243 | 1,84156 | TRUE | 0,047027 | TRUE |
| 8,624/415,17978 | 5,46E-05 | 22,4781 | TRUE | 0,000293 | TRUE |
| 9,048/315,18155 | 0,000155 | 0,874088 | TRUE | 0,000689 | TRUE |
| 9,249/387,28702 | 1,1E-12 | 1,52272 | TRUE | 5,33E-11 | TRUE |
| 9,342/399,18576 | 0,001042 | 0,260219 | TRUE | 0,003359 | TRUE |
| 9,581/236,10558 | 0,00433 | 0,845847 | TRUE | 0,009965 | TRUE |
| 9,587/293,17584 | 0,006355 | 0,860358 | TRUE | 0,013754 | TRUE |
| 9,867/399,18567 | 5,95E-05 | 0,173622 | TRUE | 0,000298 | TRUE |
| 9,867/601,32385 | 3,93E-08 | 0,79604 | TRUE | 7,13E-07 | TRUE |
| 9,879/467,17239 | 5,88E-05 | 0,18429 | TRUE | 0,000298 | TRUE |
| 9,922/343,21289 | 1,19E-09 | 0,850986 | TRUE | 3,44E-08 | TRUE |
| 9,922/399,18521 | 0,469623 | 0,784583 | FALSE | 0,508174 | FALSE |
| 9,974/399,18561 | 1,7E-05 | 7,23461 | TRUE | 0,000129 | TRUE |
| 10,006/554,26202 | 0,000629 | 0,944327 | TRUE | 0,00217 | TRUE |
| 10,37/399,18481 | 0,001789 | 0,100425 | TRUE | 0,004804 | TRUE |
| 10,425/399,18491 | 0,115092 | 0,407659 | FALSE | 0,147684 | FALSE |
| 10,521/399,18579 | 6,23E-08 | 49,3521 | TRUE | 1E-06 | TRUE |
| 10,575/399,18509 | 0,000206 | NFC | TRUE | 0,000877 | TRUE |
| 11,046/621,43701 | 0,005896 | 0,588737 | TRUE | 0,013152 | TRUE |
| 11,091/221,15454 | 0,002283 | 1,0534 | TRUE | 0,005708 | TRUE |
| 11,113/621,43719 | 3,7E-05 | 0,391666 | TRUE | 0,000244 | TRUE |
| 11,166/621,43652 | 4,39E-05 | 0,314758 | TRUE | 0,000255 | TRUE |
| 11,345/297,24319 | 0,0049 | 1,07537 | TRUE | 0,011103 | TRUE |
| 11,69/458,42099 | 5,06E-05 | 2,32462 | TRUE | 0,000282 | TRUE |
| 11,772/458,42194 | 2,09E-13 | 0,408582 | TRUE | 1,51E-11 | TRUE |
| 11,781/250,14514 | 0,122712 | 1,02394 | FALSE | 0,15339 | FALSE |

Var_ID , Variable identification; NFC= No fold change; FDR_BH,False Discovery Rate -Benjamini-Hochberg

**Table S4** CV-ANOVA for 6 versus 12 months and 6 versus 18 months harvest ages OPLSDA models from UPLC-QTOF-MS.

| **6 versus 12 months** | Sum of squires | Degrees of freedom | Mean square | F-statistic | P-value | Standard deviation |
| --- | --- | --- | --- | --- | --- | --- |
| Total correlation | 23 | 23 | 1 |  |  | 1 |
| Regression | 19.066 | 6 | 3.16776 | 13.4851 | 1.22509 ×10^-05^ | 1.77982 |
| Residual | 3.99345 | 17 | 0.234909 |  |  | 0.484674 |
| **6 versus 18 months** |  |  |  |  |  |  |
| Total correlation | 23 | 23 | 1 |  |  | 1 |
| Regression | 18.717 | 4 | 4.67924 | 20.7575 | 1.01461 ×10^-06^ | 2.16315 |
| Residual | 4.28305 | 19 | 0.225424 |  |  | 0.474788 |

**Table S5** Features that significantly influenced the OPLS-DA models comparing 6 vs 12 months and 6 vs 18 months harvest ages.

| **Name** | **Features** | **Total** |
| --- | --- | --- |
| 6vs12M and 6vs18M | 6,855/320,97009 5,964/261,13504 9,048/315,18155 5,617/787,26733 11,166/621,43652 3,028/609,12549 7,166/585,23438 5,386/173,08189 1,239/549,16864 5,991/237,04105 3,961/449,99133 9,922/343,21289 5,994/316,99786 1,226/341,10965 5,905/237,04066 7,839/215,1288 6,11/206,9937 5,295/537,19897 9,249/387,28702 10,006/554,26202 7,346/585,23474 3,094/331,10373 9,867/601,32385 6,005/554,26202 6,079/163,00394 11,345/297,24319 8,624/415,17978 8,564/415,17935 3,024/169,01474 6,053/221,04573 11,69/458,42099 6,04/163,00395 1,226/439,08325 6,847/221,04597 5,295/583,20551 10,521/399,18579 11,046/621,43701 6,079/190,99886 5,031/383,00876 2,004/554,26202 3,948/593,13147 3,29/609,12585 5,968/190,9985 5,184/383,00821 3,962/303,05124 11,772/458,42194 3,476/305,06693 9,974/399,18561 11,113/621,43719 1,224/387,11526 | 50 |
| 6vs12M | 7,291/695,40063 1,226/736,13794 1,225/440,02698 3,963/272,97141 6,855/465,08078 3,959/193,01456 1,23/377,08719 1,227/683,22571 6,847/190,99902 | 9 |
| 6vs18M | 5,829/251,0558 5,976/624,99384 3,429/272,97162 5,829/331,01318 5,195/773,25208 5,705/397,02411 8,453/401,20099 5,277/301,00323 4,006/554,26202 10,37/399,18481 9,342/399,18576 9,581/236,10558 5,974/206,02219 9,879/467,17239 3,948/404,99182 8,005/554,26202 5,975/640,96814 3,961/373,0549 5,018/270,992 8,509/415,18015 6,158/206,99416 3,383/913,18378 10,575/399,18509 9,587/293,17584 3,947/305,06738 5,978/221,04564 3,527/608,11743 3,189/913,18549 5,813/316,99716 5,909/316,99753 5,733/331,01248 9,867/399,18567 6,069/624,99457 6,156/237,04074 6,048/206,99362 3,559/609,12616 11,091/221,15454 6,138/316,99765 4,91/270,99237 3,953/611,14172 6,03/190,99915 5,465/367,01379 6,001/301,00244 5,556/242,99709 4,963/270,99228 | 45 |

**Table S6** Features that were increased and decreased from OPLS-DA model in *P. sidoides* roots samples subjected to different treatments.

| **Name** | **Features** | **Total** |
| --- | --- | --- |
| In12M In18M | 5,964/261,13504 5,617/787,26733 3,028/609,12549 5,386/173,08189 3,961/449,99133 5,905/237,04066 7,839/215,1288 6,11/206,9937 5,295/537,19897 9,249/387,28702 3,094/331,10373 6,079/163,00394 11,345/297,24319 8,624/415,17978 8,564/415,17935 3,024/169,01474 6,053/221,04573 11,69/458,42099 6,04/163,00395 6,847/221,04597 5,295/583,20551 10,521/399,18579 6,079/190,99886 5,031/383,00876 3,948/593,13147 3,29/609,12585 3,962/303,05124 5,184/383,00821 3,476/305,06693 9,974/399,18561 | 30 |
| De12M De18M | 9,048/315,18155 11,166/621,43652 7,166/585,23438 1,239/549,16864 5,991/237,04105 9,922/343,21289 5,994/316,99786 1,226/341,10965 10,006/554,26202 7,346/585,23474 9,867/601,32385 6,005/554,26202 1,226/439,08325 11,046/621,43701 2,004/554,26202 5,968/190,9985 11,772/458,42194 11,113/621,43719 1,224/387,11526 | 19 |
| In12M | 7,291/695,40063 3,963/272,97141 6,855/465,08078 3,959/193,01456 6,847/190,99902 | 5 |
| De12M | 1,226/736,13794 1,225/440,02698 1,23/377,08719 1,227/683,22571 | 4 |
| In18M | 3,429/272,97162 5,195/773,25208 5,705/397,02411 3,948/404,99182 3,961/373,0549 6,158/206,99416 3,383/913,18378 3,947/305,06738 3,527/608,11743 3,189/913,18549 5,909/316,99753 6,069/624,99457 3,559/609,12616 11,091/221,15454 6,138/316,99765 3,953/611,14172 6,03/190,99915 5,465/367,01379 5,556/242,99709 4,963/270,99228 | 20 |
| De18M | 5,976/624,99384 8,453/401,20099 4,006/554,26202 10,37/399,18481 9,342/399,18576 9,581/236,10558 5,974/206,02219 9,879/467,17239 8,005/554,26202 5,975/640,96814 8,509/415,18015 9,587/293,17584 5,978/221,04564 5,813/316,99716 5,733/331,01248 9,867/399,18567 6,048/206,99362 4,91/270,99237 6,001/301,00244 | 19 |

**Table S7** Quantification of umckalin, epigallocatechin, and structurally related compounds, expressed as umckalin and epigallocatechin equivalents.

| **Harvest age** | **Irrigation**  **(% PAW)** | **Umckalin (mg/kg DW)** | **Isofraxidin (mg/kg DW)** | **Umckalin sulphate (mg/kg DW)** | **Dihydroxy coumarin sulphate (mg/kg DW)** | **Epigallocatechin**  **(mg/kg DW)** | **Gallocatechin**  **(mg/kg DW)** | **GallocatechinD**  **(mg/kg DW)** | **EpigallocatechinD**  **(mg/kg DW)** |
| --- | --- | --- | --- | --- | --- | --- | --- | --- | --- |
| 6 Months | *75* | 260.40 ± 229.67ᵃ | 1393.24 ± 771.44ᵃᵇ | 6211.96 ± 3305.87ᵃᵇ | 295.68 ± 259.49ᵇᶜ | 342.98 ± 140.60ᵃ | 126.14 ± 39.55ᵃ | 363.47 ± 132.92ᵃ | 190.57 ± 29.24ᵃ |
|  | *50* | 169.28 ± 127.73ᵃ | 1047.49 ± 356.12ᵃᵇ | 4747.17 ± 1533.79ᵃᵇ | 150.47 ± 180.90ᶜ | 311.85 ± 147.14ᵃ | 97.66 ± 55.10ᵃ | 316.57 ± 149.51ᵃ | 149.52 ± 79.75ᵃ |
|  | *25* | 279.37 ± 279.41ᵃ | 1603.97 ± 734.33ᵃ | 7110.32 ± 3136.37ᵃ | 289.24 ± 229.61ᵇᶜ | 298.27 ± 80.74ᵃ | 67.17 ± 32.96ᵃ | 372.04 ± 115.49ᵃ | 175.12 ± 58.46ᵃ |
| 12 Months | *75* | 431.61 ± 112.94ᵃ | 1001.96 ± 259.30ᵃᵇ | 4535.17 ± 1106.33ᵃᵇ | 636.44 ± 93.53ᵃ | 505.58 ± 286.33ᵃ | 163.51 ± 121.02ᵃ | 458.92 ± 275.12ᵃ | 303.62 ± 144.06ᵃ |
|  | *50* | 388.72 ± 156.67ᵃ | 804.52 ± 247.99ᵇ | 3715.15 ± 1070.84ᵇ | 468.26 ± 215.09ᵃᵇ | 385.94 ± 152.17ᵃ | 224.60 ± 287.11ᵃ | 317.60 ± 120.16ᵃ | 247.06 ± 93.83ᵃ |
|  | *25* | 373.78 ± 351.11ᵃ | 990.06 ± 703.13ᵃᵇ | 4522.05 ± 3036.61ᵃᵇ | 317.05 ± 219.85ᵇᶜ | 344.75 ± 267.79ᵃ | 114.39 ± 144.51ᵃ | 289.88 ± 314.52ᵃ | 187.97 ± 164.06ᵃ |
| 18 Months | *75* | 197.48 ± 104.32ᵃ | 1009.11 ± 485.67ᵃᵇ | 4580.55 ± 2075.64ᵃᵇ | 321.70 ± 110.89ᵇᶜ | 479.91 ± 180.38ᵃ | 124.02 ± 79.11ᵃ | 416.01 ± 190.96ᵃ | 256.62 ± 159.12ᵃ |
|  | *50* | 251.98 ± 157.54ᵃ | 925.19 ± 244.41ᵃᵇ | 4227.00 ± 1030.92ᵃᵇ | 390.32 ± 181.97ᵃᵇᶜ | 443.52 ± 225.38ᵃ | 122.35 ± 100.46ᵃ | 459.67 ± 356.53ᵃ | 237.69 ± 130.82ᵃ |
|  | *25* | 193.09 ± 187.52ᵃ | 804.22 ± 193.79ᵇ | 3720.55 ± 828.29ᵇ | 287.34 ± 210.24ᵇᶜ | 473.16 ± 69.23ᵃ | 187.32 ± 120.26ᵃ | 428.94 ± 180.15ᵃ | 285.80 ± 27.09ᵃ |
| ANOVA Pr > F  Irrigation  Harvest  Interaction |  | 0.9535  0.0857  0.9237 | 0.5114  0.0749  0.7598 | 0.5171  0.0783  0.7675 | 0.3419  0.0322*  0.3442 | 0.6142  0.1767  0.9422 | 0.8692  0.3389  0.5986 | 0.8298  0.5943  0.8834 | 0.6576  0.1385  0.7507 |
